# Hexosamine biosynthetic pathway promotes the antiviral activity of SAMHD1 by enhancing O-GlcNAc transferase-mediated protein O-GlcNAcylation

**DOI:** 10.7150/thno.50230

**Published:** 2021-01-01

**Authors:** Jie Hu, Qingzhu Gao, Yang Yang, Jie Xia, Wanjun Zhang, Yao Chen, Zhi Zhou, Lei Chang, Yuan Hu, Hui Zhou, Li Liang, Xiaosong Li, Quanxin Long, Kai Wang, Ailong Huang, Ni Tang

**Affiliations:** 1 Key Laboratory of Molecular Biology for Infectious Diseases (Ministry of Education), Institute for Viral Hepatitis, Department of Infectious Diseases, The Second Affiliated Hospital, Chongqing Medical University, Chongqing, China.; 2 State Key Laboratory of Proteomics, Beijing Proteome Research Center, National Center for Protein Sciences (Beijing), Beijing Institute of Lifeomics, Beijing, China.; 3 School of Pharmaceutical Science, Chongqing Medical University, Chongqing, China.; 4 The First Affiliated Hospital, Chongqing Medical University, Chongqing, China.

**Keywords:** Hepatitis B virus, O-linked β-N-acetylglucosamine modification, sterile alpha motif and histidine/aspartic acid domain-containing protein 1, antiviral immunity, hexosamine biosynthetic pathway

## Abstract

**Rationale:** Viruses hijack the host cell machinery to promote viral replication; however, the mechanism by which metabolic reprogramming regulates innate antiviral immunity in the host remains elusive. Herein, we explore how the hexosamine biosynthesis pathway (HBP) and O-linked-N-acetylglucosaminylation (O-GlcNAcylation) regulate host antiviral response against hepatitis B virus (HBV) *in vitro* and* in vivo.*

**Methods:** We conducted a metabolomics assay to evaluate metabolic responses of host cells to HBV infection. We systematically explored the role of HBP and protein O-GlcNAcylation in regulating HBV infection in cell and mouse models. O-linked N-acetylglucosamine (O-GlcNAc) target proteins were identified via liquid chromatography-tandem mass spectrometry (LC-MS) and co-immunoprecipitation assays. Additionally, we also examined uridine diphosphate (UDP)-GlcNAc biosynthesis and O-GlcNAcylation levels in patients with chronic hepatitis B (CHB).

**Results:** HBV infection upregulated GLUT1 expression on the hepatocyte surface and facilitated glucose uptake, which provides substrates to HBP to synthesize UDP-GlcNAc, leading to an increase in protein O-GlcNAcylation. Pharmacological or transcriptional inhibition of HBP and O-GlcNAcylation promoted HBV replication. Mechanistically, O-GlcNAc transferase (OGT)-mediated O-GlcNAcylation of sterile alpha motif and histidine/aspartic acid domain-containing protein 1 (SAMHD1) on Ser93 stabilizes SAMHD1 and enhances its antiviral activity. Analysis of clinical samples revealed that UDP-GlcNAc level was increased, and SAMHD1 was O-GlcNAcylated in patients with CHB.

**Conclusions:** HBP-mediated O-GlcNAcylation positively regulates host antiviral response against HBV *in vitro* and* in vivo*. The findings reveal a link between HBP, O-GlcNAc modification, and innate antiviral immunity by targeting SAMHD1.

## Introduction

Immunometabolism is an emerging field that highlights the importance of specific metabolic pathways in immune regulation. Metabolic enzymes, such as glyceraldehyde 3-phosphate dehydrogenase and pyruvate kinase isozyme M2 can directly modulate immune cell activation 1,2. In addition to providing energy and building blocks for biosynthesis, metabolites have been shown to participate in epigenetic modification and signal transduction. The glycolytic product lactate not only regulates gene expression by histone acetylation 3, but also acts as a suppressor of type I interferon signaling by interacting with the mitochondrial antiviral signaling protein MAVS 4. Itaconate—another important metabolite for immune function—downregulates type I interferon signaling during viral infection by promoting alkylation of Kelch-like ECH-associated protein 1 and activation of anti-inflammatory proteins, including nuclear factor erythroid 2-related factor 2 5,6.

Viruses are obligate parasites that rely on the biosynthetic machinery of the host to complete their life cycle. On the one hand, viruses hijack the host cell machinery upon entry to fulfill their energetic and biosynthetic demands for viral replication. Human cytomegalovirus (HCMV) and herpes simplex virus-1 (HSV-1) remodel host cells to perform distinct, virus-specific metabolic programs 7. HCMV reprograms host metabolism by upregulating the expression of carbohydrate-response element binding protein and glucose transporter 4 (GLUT4) to provide materials for viral replication 8. On the other hand, hosts may recognize virus-induced signaling and reprogram metabolic pathways to protect themselves from further damage. Increased glucose utilization, increased aerobic glycolysis, and inhibition of oxidative metabolism have emerged as the hallmarks of macrophage activation 9. Pattern recognition molecules as well as several metabolic pathways and metabolites have been reported to play an important role in regulating host innate immune response 10-12. Therefore, it is important to identify the key metabolites that regulate innate immune response during viral infection.

Recent studies have emphasized the emerging role of the hexosamine biosynthesis pathway (HBP)—a branch of glucose metabolism—in host innate immunity. HBP plays a positive role in host antiviral immunity against vesicular stomatitis virus (VSV) 13, influenza virus 14, and hepatitis C virus 15. Approximately 2%-5% of the total glucose entering a cell is converted to uridine diphosphate N-acetylglucosamine (UDP-GlcNAc) 16—the end-product of HBP—and serves as a donor for O-linked β-N-acetylglucosamine (O-GlcNAc) modification (also known as O-GlcNAcylation) 17. O-GlcNAc transferase (OGT) and O-GlcNAcase (OGA) are responsible for the addition and removal of N-acetylglucosamine (GlcNAc), respectively, from Ser and Thr residues of target proteins. Several key host proteins involved in immune modulation, including signal transducer and activator of transcription-3 (STAT3), MAVS, and receptor-interacting serine/threonine-protein kinase 3 (RIPK3), are targets for O-GlcNAcylation 18,13,19,14. O-GlcNAcylation of the transcription factor STAT3 on Thr 717 negatively regulates its phosphorylation and reduces interleukin 10 (IL-10) production in macrophages 18. O-GlcNAcylation of the signaling adaptor MAVS on Ser 366 promotes retinoic -acid inducible gene-like receptor-antiviral signaling activation upon VSV infection 13. O-GlcNAcylation of kinase RIPK3 on Thr 467 suppresses inflammation and necroptosis upon LPS stimulation 19.

Sterile alpha motif and histidine/aspartic acid domain-containing protein 1 (SAMHD1) is an interferon-induced restriction factor that plays an important role in innate immune response 20. As a host deoxynucleotide triphosphate triphosphohydrolase (dNTPase), SAMHD1 degrades intracellular dNTPs to restrict viral DNA synthesis, thereby suppressing replication of diverse viruses, such as human immunodeficiency virus type 1 (HIV-1) 21 and hepatitis B virus (HBV) 22. The dNTPase activity requires homo-tetramerization of the SAMHD1 protein 23. Several post-translational modifications, including phosphorylation 24 and ubiquitination 25, have been reported to be critical for SAMHD1 function. Our group has previously demonstrated that cyclin E2-CDK2 mediates SAMHD1 phosphorylation to abrogate its restriction of HBV replication 26. However, it is unclear whether SAMHD1 can be O-GlcNAcylated by OGT and whether O-GlcNAcylation of SAMHD1 affects its antiviral function.

In this study, we investigated metabolic responses of host cells to HBV infection. We found that HBV infection induces reprogramming of host cell glucose metabolism toward the HBP pathway, and enhances O-GlcNAc modification of host proteins. Our results showed that OGT-mediated O-GlcNAcylation regulates the antiviral activity of SAMHD1. Moreover, OGT promotes O-GlcNAcylation on Ser93 to enhance SAMHD1 stability and tetramerization, which is important for its dNTPase activity. Our study identified a molecular mechanism whereby HBP/OGT-mediated O-GlcNAcylation regulates SAMHD1 function during HBV infection, highlighting the antiviral role of the HBP pathway in HBV infection.

## Materials and methods

### Cell Culture

Human hepatoma cell line HepG2 was maintained in Minimum Essential Medium (MEM, HyClone, Logan, UT, USA) supplemented with 10% fetal bovine serum (FBS, Gibco, Rockville, MD, USA), 100 U/mL penicillin, and 100 μg/mL streptomycin (HyClone). HepAD38 cells were cultured in Dulbecco's Modified Eagle Medium: Nutrient mixture F12 (DMEM /F12) containing 10% FBS, 100 U/mL penicillin, 100 μg/mL streptomycin and 400 ng/mL tetracycline (Tet) to suppress HBV pregenomic RNA (pgRNA) transcription. HEK293T and HepG2-NTCP cells were cultured in DMEM; THP-1 cells were maintained in Roswell Park Memorial Institute (RPMI) 1640 medium supplemented with 10% FBS, 100 U/mL penicillin, and 100 μg/mL streptomycin. Primary human hepatocytes (5200; Sciencell, Carlsbad, CA, USA) were purchased from Shanghai Zhong Qiao Xin Zhou Biotechnology Co., Ltd. and maintained in hepatocyte medium (5201; Sciencell).

### Animal models

HBV-transgenic (HBV-Tg) mice (n = 6 for each group) were kindly provided by Prof. Ning-shao Xia, School of Public Health, Xiamen University 27. C57BL/6J mice (6- to-8-week-old, six per group) were provided by the Laboratory Animal Center of Chongqing Medical University (SCXK (YU) 2018-0003). Mice were intraperitoneally injected with 6-diazo-5-oxo-L-norleucine (DON; 1 mg/kg body weight), Thiamet G (TMG; 20 mg/kg body weight), or phosphate-buffered saline (PBS) as a control every other day for 10 times. On day 20 post-administration, mouse serum and liver tissue specimens were collected for real-time PCR, Southern blotting, and immunohistochemical staining. Mice were treated in accordance with the guidelines established by the Institutional Animal Care and Use Committee at the Laboratory Animal Center of Chongqing Medical University. The animal care and use protocols adhered to the National Regulations for the Administration of Laboratory Animals to ensure minimal suffering.

### Clinical samples

The study protocol was approved by the Medical Ethics Committee of Chongqing Medical University. Informed consent was obtained from patients who met the inclusion criteria for chronic HBV infection. Clinical samples of patients were collected from volunteers during physical examinations between May 2019 and December 2019 at the Second Affiliated Hospital of Chongqing Medical University. Blood samples or liver tissue were obtained from patients (cohort #1: 21 males and 25 females, aged 30.76 ± 8.35 years; cohort #2: 4 males, aged 27 ± 4.08 years) confirmed to be infected by HBV. Blood samples or liver tissue from healthy individuals (cohort #1: 37 males and 13 females, aged 34.06 ± 5.55 years; cohort #2: 1 males and 3 females, aged 52.25 ± 19.07 years) were randomly selected as controls from the Second Affiliate Hospital of Chongqing Medical University. In this study, patients with HBV infection were confirmed as being infected with HBV virus via qPCR or ELISA.

### Plasmids and molecular cloning

The adenoviral vector Ad-HBV1.3 was a kind gift from Prof. Michael Nassal (University Hospital Freiburg, Freiburg, Germany). The expression of the complete HBV genome was confirmed to ensure successful completion of HBV replication. The cloned human vector cDNAs for SAMHD1 and OGT were ligated into the pSEB-3xFlag expression vector to produce Flag-tagged proteins. The Ser-Ala and Thr-Glu mutants of SAMHD1 (S93A and T592E, respectively) were constructed by site-directed mutagenesis using wild-type (WT) SAMHD1-3xFlag plasmid as a template. The recombinant prokaryotic vector for His-tagged human SAMHD1 expression was constructed by subcloning PCR-amplified inserts into the N-terminal 6× His-tagged pET-28a vector. HA-tagged OGT plasmid was constructed by insertion of OGT cDNA into the pBudCE-3xHA vector. All constructs were confirmed via DNA sequencing and western blotting. Oligonucleotide sequences used for cloning are listed in Table S1.

### Lentivirus-mediated small hairpin RNA (shRNA) interference

Lentiviruses were generated as described previously 28. Briefly, double-stranded (shRNA) sequences targeting OGA, OGT, and GFPT1 were synthesized, annealed, and inserted into the *Hpa*I and *Xho*I sites of pLL3.7 lentiviral vector (kindly provided by Prof. Bing Sun, Shanghai Institute of Biochemistry and Cell Biology, Chinese Academy of Sciences). The VSVG, Δ8.9, and pLL3.7 vectors containing shRNA sequences targeting OGA (sh*OGA*), OGT (sh*OGT*), GFPT1 (sh*GFPT1*), respectively, or a scrambled control (shCon) were co-transfected into HEK293T cells using Lipofectamine 2000 reagent (Thermo Fisher Scientific, Waltham, MA, USA), following the manufacturer's instructions. HepG2 or HepAD38 cells were transduced with the packaged lentiviruses in the presence of polybrene (5 μg/mL).

### CRISPR/Cas9 system

The CRISPR/Cas9 plasmids lentiCRISPR-v2, pMD2.G, and psPAX2 were kindly provided by Prof. Ding Xue, School of Life Sciences, Tsinghua University, Beijing, China. To generate SAMHD1 or OGT knockout (KO) cells, single guide RNA (sgRNA) sequences were cloned into the lentiCRISPR-v2 vector (Table S1). Lentivirus was generated by co-transfecting HEK293T cells with lentiCRISPR-v2, the envelope expressing plasmid pMD2.G, and the packaging plasmid psPAX2 using Lipofectamine 2000 (Invitrogen, Carlsbad, CA, USA), following the manufacturer's instructions. HepG2 and HepAD38 cells were subsequently infected with the filtered lentiviral supernatant and selected with puromycin (1.5-2.0 μg/mL, ab141453, Abcam, Cambridge, UK). The single-cell HCC clones stably expressing sgRNA were propagated and validated using immunoblotting and DNA sequencing.

### Quantitative reverse transcription-PCR (qRT-PCR)

Total RNA was extracted from human peripheral blood mononuclear cells, HepG2, and HepAD38 cells, and reverse-transcribed into cDNAs using PrimeScript™ RT Reagent Kit with gDNA Eraser (RR047A, TaKaRa, Tokoyo, Japan). Quantitative real-time PCR analysis of target genes was performed using the SYBR Green qPCR Master Mix (Bio-Rad, Hercules, CA, USA) with specific primers (Table S1). The fold difference in mRNA expression between various treatment groups was determined using the standard ΔΔCt method; glyceraldehyde 3-phosphate dehydrogenase (*GAPDH*) was used as an internal control. All samples were analyzed in triplicate, and the data represent at least three independent experiments.

### Quantification of HBV DNA levels using real-time PCR

Quantitative real-time PCR was performed to determine HBV DNA levels. Cells and liver tissues were lysed in lysis buffer (1 mM EDTA; 10 mM Tris-HCl, pH 8.0; 2% sucrose; and 1% NP-40) and subsequently treated with micrococcal nuclease and 10 mM CaCl_2_ for 1 h at 37 °C. Viral DNA was precipitated using 35% polyethylene glycol (PEG) 8000 and digested overnight with 0.5 mg/mL proteinase K at 45 °C. Nucleic acids were purified, and the precipitated DNA was quantified using quantitative PCR 29. The plasmid pCH9/3091 (containing 1.1 copies of the HBV genome) served as a template for the standard curve.

### Southern blotting

Southern blotting was performed as described previously 30. Briefly, extracted DNA samples were electrophoresed on a 1.2% agarose gel. The separated DNA fragments were denatured in 0.5 M NaOH and 1.5 M NaCl, neutralized in 1 M Tris-HCl (pH 7.4) and 1.5 M NaCl, and transferred onto a nylon membrane. The membrane was UV cross-linked, and HBV DNA was detected after hybridization with a digoxigenin (DIG)-labeled full-length HBV genome probe (DIG high prime DNA labeling and detection starter kit, Roche Diagnostics GmbH, Mannheim, Germany).

### Immunoblotting

Cell lysates and the liver tissue homogenate were denatured in 6× SDS-PAGE loading buffer, separated by 10% SDS-PAGE, and electro-transferred to a PVDF membrane (Millipore, Billerica, MA, USA). The immunoblots were probed with the indicated antibodies (Table S2). Protein bands were visualized using SuperSignal™ West Pico Chemiluminescent Substrate kits (Bio-Rad) and quantitated by densitometry using Image J software.

### Immunohistological staining

Liver tissue samples were fixed in 4% paraformaldehyde and embedded in paraffin according to standard procedures. Sections were incubated with the indicated primary antibodies overnight at 4 °C. Thereafter, the slides were incubated with a secondary anti-rabbit or anti-mouse IgG antibody (ZSGB-BIO, Beijing, China) and visualized using 3,3'-diaminobenzidine (ZSGB-BIO). Stained slides were scanned with a Pannoramic Scan 250 Flash or MIDI system, and images were acquired using Pannoramic Viewer 1.15.2 (3DHistech, Budapest, Hungary).

### Immunoprecipitation (IP) assay

HEK293T, SAMHD1-KO HepG2, or SAMHD1-KO HepAD38 cells were transfected with Flag-tagged SAMHD1-WT or -mutant expression constructs for 48 h. Cells were resuspended in lysis buffer (50 mM Tris HCl, pH 7.4; 150 mM NaCl; 1 mM EDTA; 1% Triton X-100) containing protease (Roche) and phosphatase (Beyotime Biotech, Shanghai, China) inhibitor cocktail. Pre-cleared lysates were incubated overnight with anti-Flag M2 affinity gel (A2220, Sigma, St. Louis, MO, USA) at 4 °C. To detect SAMHD1-mediated ubiquitination, HEK293T cells were first co-transfected with HA-Ubiquitin and Flag-tagged SAMHD1 WT or S93A mutant, and subsequently treated with TMG. The endogenous K48 ubiquitination of SAMHD1 was detected by two step immunoprecipitation (Re-IP) after boiling and probing for ubiquitination. Briefly, the cell extracts were immunoprecipitated with SAMHD1 antibody. The beads were washed and then heated at 98 °C in 1% SDS in order to disrupt noncovalent protein-protein interactions. The immunoprecipitates were re-immunoprecipitated (Re-IP) with SAMHD1 antibody and then subjected to immunoblotting analysis.

### Metabolites analysis

To extract metabolites from quenched serum/plasma samples or cell culture supernatants, 400 μL chilled methanol: acetonitrile (2:2, v/v) was added to 100 μL of each sample. The mixture was vortexed three times for 1 min each, with 5 min incubation at 4 °C after each vortexing step. After the final vortexing step of 30 s, the mixture was incubated on ice for 10 min. Thereafter, 100 μL chilled HPLC-certified water was added to the samples, mixed for 1 min, and centrifuged at 13,000 × *g* for 10 min at 4 °C. Finally, the liquid phase (supernatant) of each sample was transferred into a new tube for UHPLC-QTOF-MS analysis at Shanghai Applied Protein Technology Co., Ltd. UDP-GlcNAc and glucose were quantified using targeted (LC-MS/MS). The data acquisition, principal component analysis, heatmap, and pathway impact analysis were performed by Shanghai Applied Protein Technology Co., Ltd.

### IP assay coupled with mass spectrometry (IP-MS)

HepAD38 (Tet-off) cell lysates were incubated overnight with an anti-O-GlcNAc antibody at 4 °C, followed by 4 h incubation with protein A/G agarose beads. Immunoprecipitated complexes were eluted and stained with Coomassie blue. Stained protein bands were sent to Shanghai Applied Protein Technology Co., Ltd for identification of potential O-GlcNAc-modified proteins. Protein bands were dissolved in 1 mL chilled methanol: acetonitrile: H_2_O (2:2:1, v/v/v) and sonicated at low temperature (30 min); this process was repeated twice. The supernatant was dried in a vacuum centrifuge. For LC-MS analysis, samples were re-dissolved in 100 μL acetonitrile: water (1:1, v/v). Sample analyses were performed using a UHPLC system (1290 Infinity LC, Agilent Technologies, Santa Clara, CA, USA) coupled to a quadrupole time-of-flight analyzer (TripleTOF 6600, AB Sciex, Toronto, Canada) at Shanghai Applied Protein Technology Co., Ltd.

### SAMHD1 O-GlcNAcylation site mapping

MS was performed to identify SAMHD1 O-GlcNAcylation sites, as described previously 31. Briefly, immunoprecipitated SAMHD1 from HEK293T cells was subjected to SDS-PAGE. The band corresponding to SAMHD1 was excised, digested overnight with trypsin, and subjected to LC-MS/MS analysis. An online LC-MS/MS setup consisting of an Easy-nLC system and an Orbitrap Fusion Lumos Tribrid mass spectrometer (Thermo Fisher Scientific GmbHThermo Scientific, Dreieich, Germany) equipped with a nanoelectrospray ion source was used for all LC-MS/MS experiments. Raw MS files were searched against the UniProt database using the MaxQuant software (version 1.5.2.8). The fixed modification was set to C (carbamidomethyl), and the variable modifications were set to M (oxidation), protein N-term (acetyl), and S/T (O-GlcNAc). The peptide tolerance for the first search was set at 20 ppm and that for the main search was set at 6 ppm. The MS/MS tolerance was 0.02 Da. The false discovery level in protein sequence motif (PSM) and protein was 1%. The match between runs was used and the minimum score for modified peptides was set at 40.

### SAMHD1 oligomerization assay

SAMHD1-KO HepG2 or HepAD38 cells were transfected with Flag-tagged SAMHD1-WT or S93A mutant vectors for 48 h. Cells were lysed with HEPES lysis buffer (40 mM HEPES, pH 7.5; 150 mM NaCl; 0.1% NP-40) containing protease (Roche) and phosphatase (Beyotime) inhibitor cocktails. Whole-cell lysates (3 mg/mL) were cross-linked with 0.025% glutaraldehyde for 5 min at 37 °C and quenched with 50 mM Tris-HCl, pH 8.0. The samples were then separated on a 4%-20% precast gel and analyzed by through western blotting using the indicated antibodies.

### Immunofluorescence microscopy

GLUT1 was detected using a polyclonal rabbit anti-GLUT1 antibody (Abcam, ab115730) in HepG2, HepG2-NTCP, and HepAD38 cells. Specific signals were visualized using Alexa Fluor 488 secondary antibody (Invitrogen). For nuclear staining, cells were treated with 1 μg/mL DAPI (10236276001, Roche) for 3 min. Stained sections were analyzed using a laser scanning confocal microscope (Leica TCS SP8, Solms, Germany).

### Detection of HBV DNA and HBV antigen

Cell culture supernatants and mouse serum HBV DNA were extracted using the TIANamp Virus DNA/RNA Kit (DP315, Tiangen, Beijing, China) and quantified via qPCR using SYBR Green dye. Hepatitis B e-antigen (HBeAg) levels were quantified using ELISA (Kehua Bio-Engineering, Shanghai, China) following the manufacturer's instructions.

### Succinylated wheat germ agglutinin (sWGA) pull-down assay

Hepatic cells and liver tissues were lysed in a buffer containing 125 mM NaCl; 50 mM Tris, pH 7.4; 5 mM EDTA; and 0.1% NP-40. The lysate was denatured in glycoprotein-denaturing buffer and digested with PNGase F (P0704S, New England Biolabs, Ipswitch, MA, USA) to remove N-linked glycoproteins. Pre-cleared lysates were incubated overnight with sWGA-conjugated agarose beads (Vector Laboratories, Burlingame, CA, USA). Precipitated complexes were eluted and immunoblotted with the indicated antibodies.

### Determination of SAMHD1 stability using cycloheximide chase assay

HepAD38 cells were first transfected with SAMHD1-WT or -mutant plasmid. The transfected cells were subsequently treated with 100 μM cycloheximide and harvested at 0, 24, 48, and 72 h. Cells were lysed, and SAMHD1 was detected using an anti-Flag antibody (Sigma).

### SAMHD1 expression and purification

His-tagged pET28a-SAMHD1 constructs were expressed in *Escherichia coli* BL21 (DE3), and expression was induced with 0.2 mM isopropyl β-D-1-thiogalactopyranoside (IPTG). Cells were resuspended in a buffer containing 20 mM Tris, pH 8.0; 300 mM NaCl; and 10 mM imidazole and lysed by sonication for 30 min. Cell lysates were purified using Ni-NTA affinity column (GE Healthcare, Chicago, IL, USA). SAMHD1 was eluted in 20 mL elution buffer (20 mM Tris-HCl, pH 8.0; 300 mM NaCl; 400 mM imidazole).

### Determination of SAMHD1 dNTPase activity using HPLC

The dNTPase assay was conducted in a reaction buffer containing 20 mM Tris-HCl, pH 7.8; 50 mM NaCl; and 5 mM MgCl_2_, in the presence of 500 μM dNTPs and 500 μM protein sample. Reactants were incubated for the indicated time at 25 °C, and the reaction was terminated by adding 10 mM EDTA. SAMHD1 was purified by centrifugation at 12,000 × *g* for 20 min with an Amicon Ultra 0.5 mL 10 kDa filter (Millipore). Nucleotide hydrolysis reactions were analyzed using a Venus ilMP-C18 (150 × 4.6 mm) column (Agela Technologies, Tianjin, China) on a Waters HPLC system. Injected samples were eluted with an 8-min linear gradient of 0% to 35% methanol (buffer B), followed by an isocratic phase of 35% buffer B over 10 min at a flow rate of 1 mL/min. The absorbance data at 260 nm were used to quantify the DNA product.

### HIV-1 NL4-3 luciferase assay

To prepare the VSV-G-pseudotyped HIV-1 single-round luciferase virus (HIV-1-LUC-G), HEK293T cells were co-transfected with 10 μg pNL4-3.Luc.R-E 32 and 2 μg VSVG. The supernatant (infectious virus) was harvested at 48-72 h post-transfection and filtered through a 0.45 μm filter. The pseudotyped viruses were used to infect differentiated SAMHD1-KO THP-1 cells (MOI 1-10). After 48 h of infection, cells were lysed with 50 μL passive lysis buffer (Promega, Madison, WI, USA). The relative luminescence units (RLUs) of luciferase were quantitated using the Luciferase Assay kit (Promega) and normalized for protein concentration.

### HBV infection in HepG2-NTCP and PHH cells

HBV virus particle collection and infection in HepG2-NTCP or in primary human hepatocyte (PHH) cells were conducted as described previously 26,33. Briefly, HepG2-NTCP cells were inoculated with concentrated HBV viral particles derived from supernatants of HepAD38 cells at 1000 genome equivalents (GE) per cell, in medium containing 5% PEG 8000 for 16 h; thereafter, the virus was removed from infected cells, and the cells were maintained in Williams' E media before harvest.

PHH cells were treated with purified HBV particles at 1000 GE per cell. To increase infectivity and virus-cell contact, the medium was supplemented with 4% PEG 8000. After 24 h, cells were washed three times with PBS and cultured in PHH maintenance medium. The culture media was changed every second day.

### Statistical Analysis

All data were expressed as the mean ± standard deviation (SD). All statistical analyses were performed using GraphPad Prism 5.0 software (GraphPad Software Inc.). Statistical significance was determined using one-way ANOVA for multiple comparisons. Student's *t*-test was used to compare two groups. *P* < 0.05 was considered statistically significant.

## Results

### HBV infection upregulates GLUT1 expression and enhances HBP activity and protein O-GlcNAcylation

**Figure 1:**
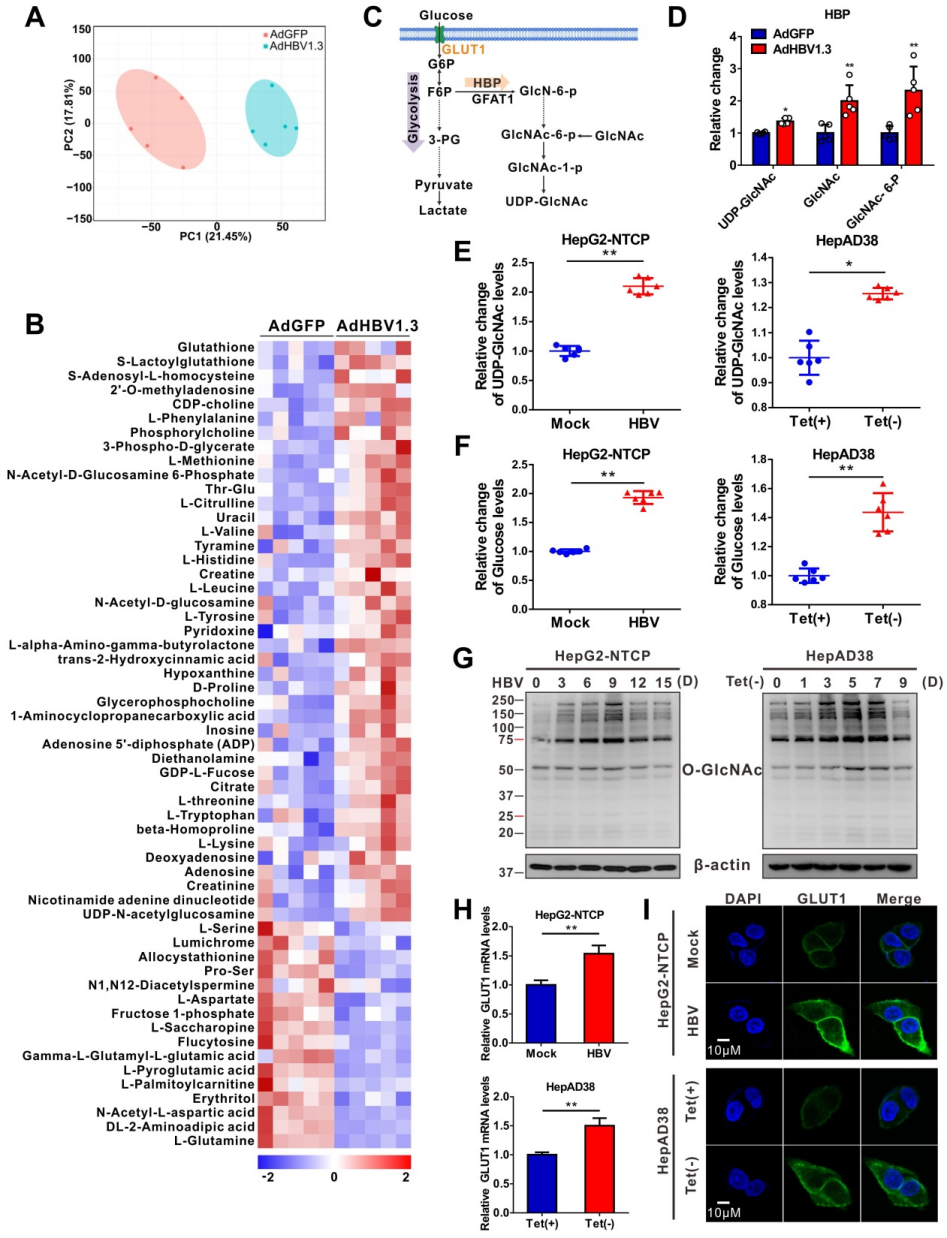
** HBV infection promotes HBP and increases protein O-GlcNAcylation.** (**A**) Principal component analysis of metabolite profiles obtained using a metabolomics assay in HepG2 cells infected with AdHBV1.3 or AdGFP for 72 h. (**B**) Heatmap of differentially expressed metabolites subjected to identical treatment conditions as in (A). (**C**) An overview of the hexosamine biosynthesis pathway (HBP). (**D**) Relative changes in intermediate metabolites of HBP. (**E**-**F**) Relative changes in the levels of UDP-GlcNAc (E) and glucose (F) in HBV-infected HepG2-NTCP cells and HepAD38 cells with tetracycline inducible (Tet-off) HBV expression was determined using the LC-MS/MS targeted metabolomics assay. (**G**) Immunoblot of total O-GlcNAc from HepG2-NTCP and HepAD38 cells treated for the indicated periods. (**H**-**I**) qPCR quantification, n = 3 (H) and immunofluorescence staining (I) of GLUT1 in HepG2-NTCP and HepAD38 cells, DAPI (blue) was used to counterstain nuclei, Scale bar, 10 μm. Data are expressed as the mean ± SD. *P* values were derived from unpaired, two-tailed Student's *t*-test in E, F, and H (*P < 0.05,**P < 0.01).

To explore metabolic changes in response to HBV infection, a metabolomics assay was performed in AdHBV-1.3-infected HepG2 cells (HepG2-HBV1.3) and AdGFP-infected HepG2 cells (HepG2-GFP). Principal component analysis showed that HBV infection dramatically changed the intracellular metabolic profile of HepG2 cells (Figure 1A). Several metabolic pathways, including central carbon, amino sugar, and nucleotide sugar metabolism (Figure S1A), were significantly affected. Recent studies have shown that glucose metabolism plays a key role in host antiviral immunity 13,14. Hence, we determined the effect of altering glucose metabolism in HepG2-HBV1.3 cells. The expression level of several intermediate metabolites in glucose metabolism, including 3-phospho-glycerate, GlcNAc, N-acetyl glucosamine 6-phosphate (GlcNAc-6-P), and UDP-GlcNAc—the end-product of HBP—was increased upon HBV infection (Figure 1B-D). To confirm this result, we established a strain of HepG2 cells engineered to express the human solute carrier family 10 member 1 (*SLC10A1*, also called *NTCP*) gene (HepG2-NTCP cells) to support HBV infection 34. Targeted liquid chromatography-tandem MS (LC-MS/MS) results showed a significant increase in UDP-GlcNAc and glucose levels in HBV-infected HepG2-NTCP, stable HBV-expressing HepAD38 (a tetracycline (Tet)- inducible HBV expression cell line) (Figure 1E-F), and AdHBV-1.3-infected HepG2 (Figure S1B-C) cells. These results were consistent with those observed in HepG2.2.15, an HBV-replicating cell line 35. HBP links cellular glucose, glutamine, acetyl-CoA, and uridine triphosphate (UTP) concentrations with signal transduction pathways through protein O-GlcNAcylation 36. Because OGT-mediated protein O-GlcNAcylation is highly dependent on the intracellular concentration of the donor substrate UDP-GlcNAc, we examined whether HBV infection can affect O-GlcNAc modification in host cells. Total protein O-GlcNAcylation in HBV-infected HepG2-NTCP cells significantly increased 6 to 9 days post HBV infection. A similar result was observed in HepAD38 (Tet-off) cells (3 to 7 days after Tet removal from the medium) (Figure 1G) and HBV-infected PHH cells (4 to 8 days post HBV infection) (Figure S1D). GLUT1 is the main regulator of glucose influx, and transcriptome analysis has shown that it is upregulated upon HBV infection 37. Consistently, GLUT1 expression was markedly enhanced in our HBV cell models (Figure 1H-I, S1E-F). Elevated glucose levels can increase HBP flux and enhance UDP-GlcNAc synthesis 38. We did not observe significant changes in the protein levels of OGT, OGA, and GFPT1—the key enzymes that regulate HBP flux and protein O-GlcNAcylation (Figure S1G-H). These findings demonstrate that HBV infection upregulates GLUT1 expression, promotes glucose uptake, and increases UDP-GlcNAc synthesis and protein O-GlcNAcylation in host cells.

### Inhibition of protein O-GlcNAcylation promotes HBV replication in host cells

**Figure 2:**
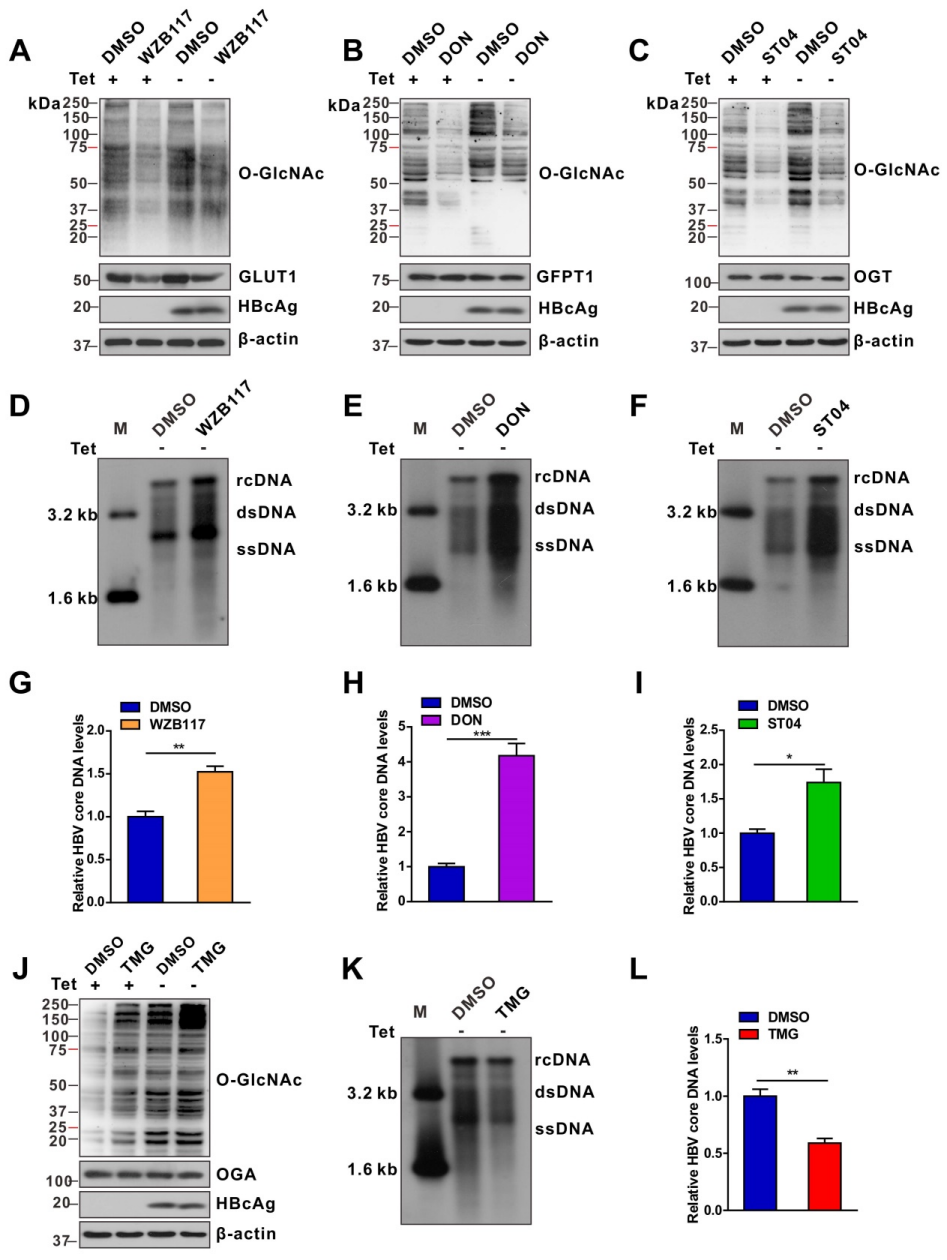
** Pharmacological inhibition of protein O-GlcNAcylation promotes HBV replication.** (**A**-**C**) Immunoblot of total O-GlcNAc from tetracycline-inducible HepAD38 cells treated with or without GLUT1 inhibitor WZB117 (50 μM) (A), GFPT1 inhibitor DON (30 μM) (B), or OGT inhibitor ST04 (100 μM) (C) for 72 h. DON, 6-Diazo-5-oxo-L-norleucine; ST04, ST045849. (**D**-**F**) HBV DNA was detected via Southern blotting assay in stable HBV-expressing HepAD38 cells treated as described above. rc DNA, relaxed circular DNA; ds DNA, double-stranded DNA; ss DNA, single-stranded DNA. (**G**-**I**) Quantification of HBV core DNA levels in stable HBV-expressing HepAD38 cells treated as indicated using qPCR, n = 3. (**J**) Immunoblot of total O-GlcNAc from tetracycline-inducible HepAD38 cells treated with or without the OGA inhibitor TMG (100 μM) for 72 h. TMG, Thiamet G. (**K**-**L**) Southern blotting analysis of HBV DNA and qPCR quantification of HBV core DNA levels in stable HBV-expressing HepAD38 cells treated as in (J), n = 3. Data are expressed as the mean ± SD. *P* values were derived from unpaired, two-tailed Student's *t*-test in G-I and L; (*P < 0.05,**P < 0.01,***P < 0.001).

**Figure 3:**
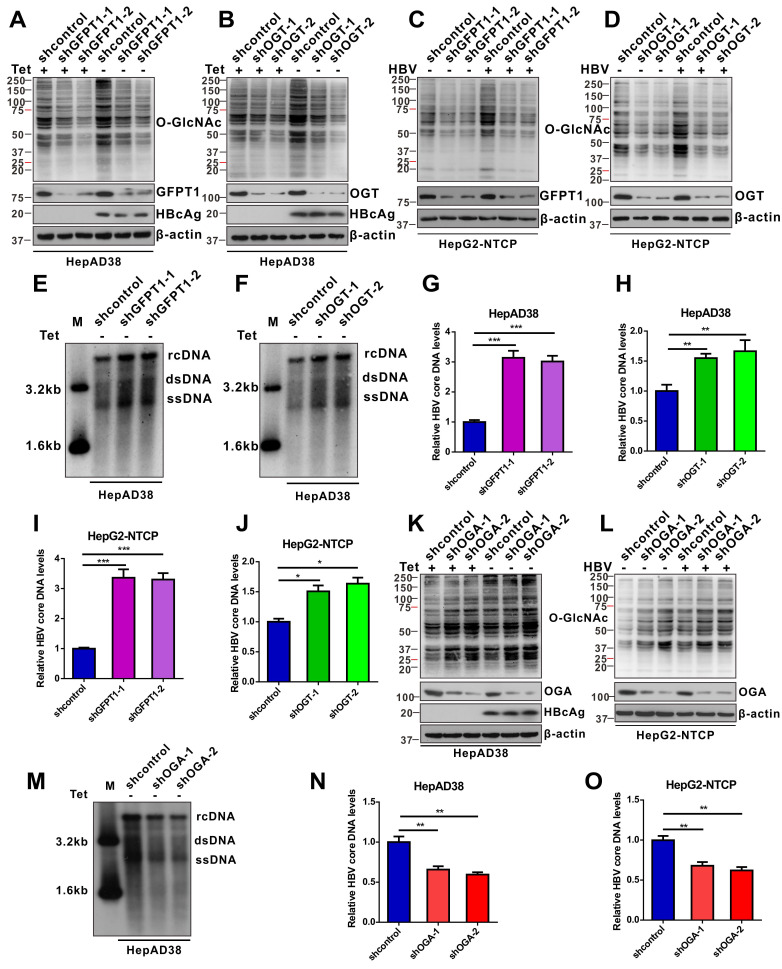
** shRNA-mediated inhibition of protein O-GlcNAcylation enhances HBV replication.** (**A**-**D**) Immunoblot of total O-GlcNAc from tetracycline-inducible HepAD38 cells (Tet-off) (A-B) and HBV-infected HepG2-NTCP cells (C-D) following shRNA-mediated knockdown of GFPT1 and OGT. (**E**-**H**) Southern blotting analysis of HBV DNA (E-F) and qPCR quantification of HBV core DNA levels (G-H) in stable HBV-expressing HepAD38 cells treated as above, n = 3. (**I**-**J**) Quantification of HBV core DNA levels in HBV-infected HepG2-NTCP cells treated as indicated using qPCR, n = 3. (**K**-**L**) Immunoblot of total O-GlcNAc from OGA-knockdown HepAD38 (Tet-off) cells (K) and OGA-knockdown HBV-infected HepG2-NTCP cells (L). (**M**) Southern blotting analysis of HBV DNA in stable HBV-expressing HepAD38 cells of OGA-knockdown. (**N**-**O**) Quantification of HBV core DNA levels in stable HBV-expressing HepAD38 cells (N) and HBV-infected HepG2-NTCP cells; (O) qPCR of HBV DNA in stable HBV-expressing HepAD38 cells of OGA-knockdown, n = 3. Data are expressed as the mean ± SD. *P* values were derived from one-way ANOVA in G-H, I-J, and N-O; (*P < 0.05, **P < 0.01, ***P < 0.001).

We evaluated the effects of protein O-GlcNAcylation on HBV replication. HepAD38 (Tet-off) cells, HepG2-HBV1.3 and HBV-infected HepG2-NTCP cells were treated with inhibitors of GLUT1, GFPT1, OGT, and OGA. Pharmacological inhibition of GLUT1, GFPT1, and OGT reduced total protein O-GlcNAcylation levels (Figure 2A-C, S2A-C, and S3A-E) and promoted HBV replication (Figure 2D-I, S2D-F, and S3F-J). Conversely, Thiamet G (TMG), a pharmacological inhibitor of OGA, increased protein O-GlcNAcylation levels (Figure 2J, S2G, and S3K-L) but suppressed HBV replication (Figure 2K-L, S2H, S3M-N). These data suggest that HBP-mediated O-GlcNAcylation negatively regulates HBV DNA replication but does not affect the protein synthesis of hepatitis B core antigen (HBcAg), OGA, OGT and GFPT1 (Figure 2 and S2). The results of pharmacological inhibitor studies were similar to those obtained from shRNA-mediated knockdown of *GFPT1*, *OGT*, or *OGA* in HepAD38 (Tet-off), HBV-infected HepG2-NTCP, and HepG2-HBV1.3 cells (Figure 3 and S4). Taken together, these results indicate that inhibition of HBP or protein O-GlcNAcylation promotes HBV DNA replication, whereas increased O-GlcNAc modifications suppress HBV DNA replication.

### OGT mediates O-GlcNAcylation of SAMHD1 upon HBV infection

**Figure 4:**
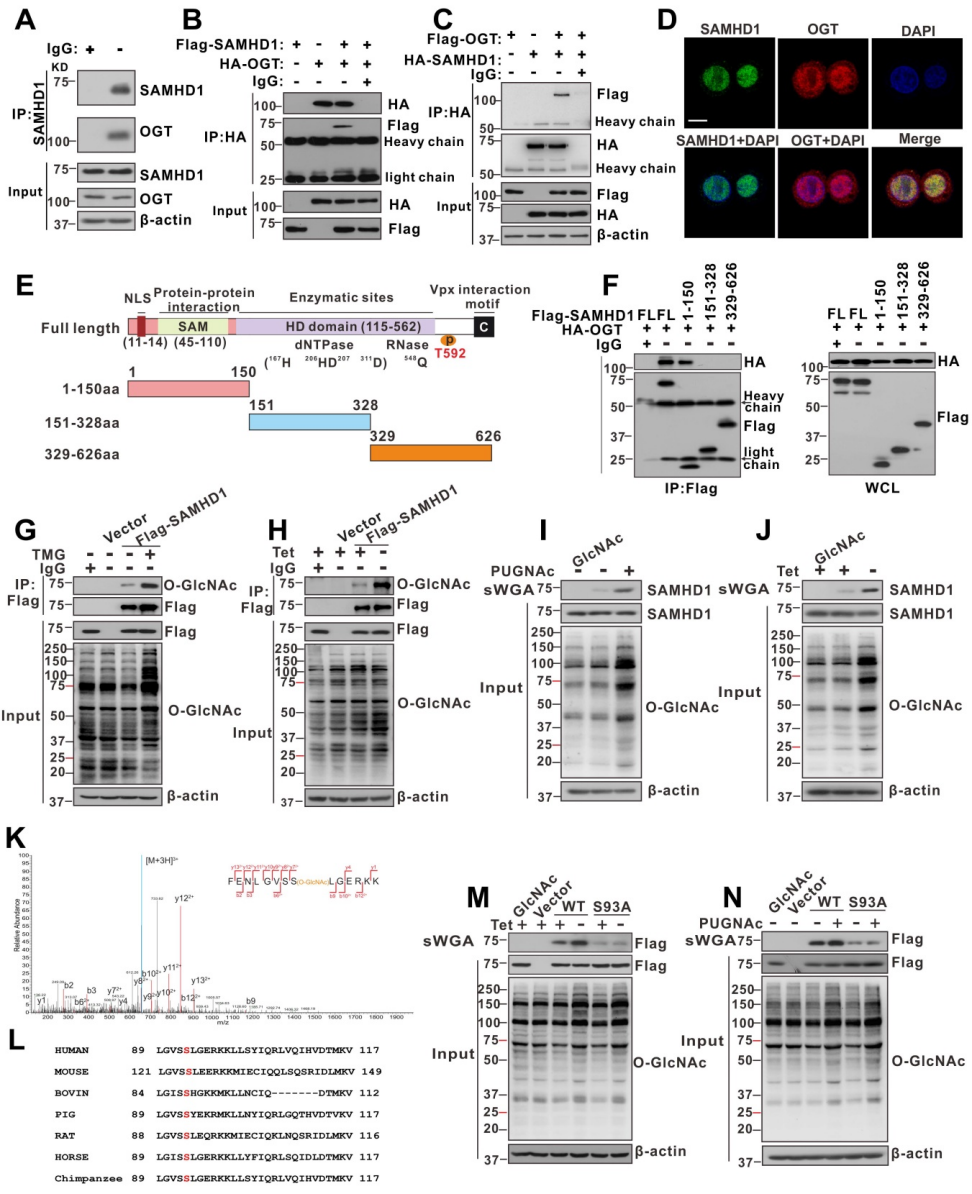
** OGT mediates O-GlcNAcylation of SAMHD1 on Ser93.** (**A**) Physical interaction between endogenous SAMHD1 and OGT. Cell extracts were immunoprecipitated with anti-SAMHD1 antibody. The immunoprecipitated and input proteins were probed with the indicated antibodies. (**B**) Immunoprecipitation (IP) of OGT with anti-HA antibody in HEK293T cells co-transfected with Flag-SAMHD1 and HA-OGT expression constructs; bands of heavy chain and light chain in the IP results were labeled. (**C**) IP of SAMHD1 with anti-HA antibody in HEK293T cells co-transfected with HA-SAMHD1 and Flag-OGT expression constructs; bands of heavy chain and light chain in the IP results were labeled. (**D**) Immunofluorescence staining of HepG2 cells using monoclonal anti-SAMHD1 (green) and anti-OGT (red) antibodies to detect their endogenous localization. DAPI (blue) was used to counterstain nuclei. Representative images are shown; scale bar: 10 μm. (**E**-**F**) The interaction between OGT and the full-length or the truncated SAMHD1 (1-150 aa, 151-328 aa, 329-626 aa), as indicated in the diagram (NLS, nuclear localization signal) (E), were determined by Co-IP in HEK293T cells (FL, full-length) (F). (**G**) HEK293T cells were transfected with the Flag-SAMHD1 construct and the control vector for 48 h and treated with 100 μM Thiamet G (TMG) for 12 h. Following cell lysis, SAMHD1 was immunoprecipitated using anti-Flag M2 agarose beads. The immunoprecipitated and input proteins were probed with an anti-O-GlcNAc or anti-Flag antibody. (**H**) Immunoprecipitation of SAMHD1 with anti-Flag M2 agarose beads in tetracycline-inducible HepAD38 cells transfected with Flag-SAMHD1 and the control vector. (**I**-**J**) HEK293T cells (I) were treated with PUGNAc, and tetracycline-inducible HepAD38 cells (J) were treated as in (H). After cell lysis, O-GlcNAc-modified proteins were purified using succinylated wheat germ agglutinin (sWGA)-conjugated agarose beads and probed with an anti-Flag or anti-O-GlcNAc antibody. GlcNAc served as a negative control. (**K**) LC-MS/MS analysis of Flag-tagged SAMHD1 identified Ser93 as the SAMHD1 O-GlcNAcylation site. Tandem MS spectrum of the +2 ion at m/z 508.97 corresponding to O-GlcNAcylated SAMHD1 peptide FENLGVSSLGERKK is shown. (**L**) Multiple sequence alignment of SAMHD1 in different species. (**M**-**N**) SAMHD1-KO HepAD38 cells were transfected with an empty vector, Flag-tagged SAMHD1-WT or S93A mutant (M). HEK293T cells were transfected with the above plasmids described in (M) and treated with 100 μM PUGNAc for 12 h (N). Cell lysates were purified using sWGA-conjugated agarose beads and probed with an anti-Flag or anti-O-GlcNAc antibody.

To further investigate the mechanism by which OGT-mediated protein O-GlcNAcylation inhibits HBV infection, we screened putative O-GlcNAc-modified proteins in HepAD38 (Tet-off) cells using the IP-MS. Cell lysates were immunoprecipitated with O-GlcNAc antibodies and analyzed by LC-MS/MS. A total of 1,034 candidate O-GlcNAc-modified proteins were identified (Table S3). Gene ontology analysis showed that several proteins were involved in innate immune and inflammatory responses (Figure S5A). We next focused on SAMHD1, which plays an important role in promoting host antiviral innate immunity 39. Interactions between OGT and SAMHD1 were demonstrated via co-immunoprecipitation (co-IP) experiments in HepG2 cells (Figure 4A) andHEK293T cells (Figure 4B-C). Confocal analysis indicated that endogenous OGT and SAMHD1 are co-localized in the nucleus (Figure 4D). We subsequently constructed three SAMHD1 deletion mutants (Figure 4E) and showed that the SAM domain of SAMHD1 is required for its interaction with OGT (Figure 4F). Immunoprecipitated Flag-tagged SAMHD1 exhibited a strong O-GlcNAc modification signal in HEK293 cells upon treatment with the OGA inhibitor TMG (Figure 4G). Meanwhile, HBV replication enhanced SAMHD1 O-GlcNAcylation in HepAD38 (Tet-off) cells (Figure 4H). These results were further confirmed in HBV-infected HepG2-NTCP cells and HBV-infected PHH cells by affinity chromatography using succinylated wheat germ agglutinin (sWGA), a modified lectin that specifically binds O-GlcNAc-containing proteins (Figure 4I-J, S5B-E). These data indicate that SAMHD1 interacts with and can be O-GlcNAcylated by OGT upon HBV infection. Moreover, TMG treatment or shRNA-mediated OGA knockdown reduced HBV replication in parental cells but failed to moderate HBV replication in SAMHD1-KO cells, indicating that TMG treatment or OGA knockdown decreased HBV replication in an SAMHD1-dependent manner (Figure S5F-G).

### OGT-mediated O-GlcNAcylation on Ser93 enhances SAMHD1 stability

**Figure 5:**
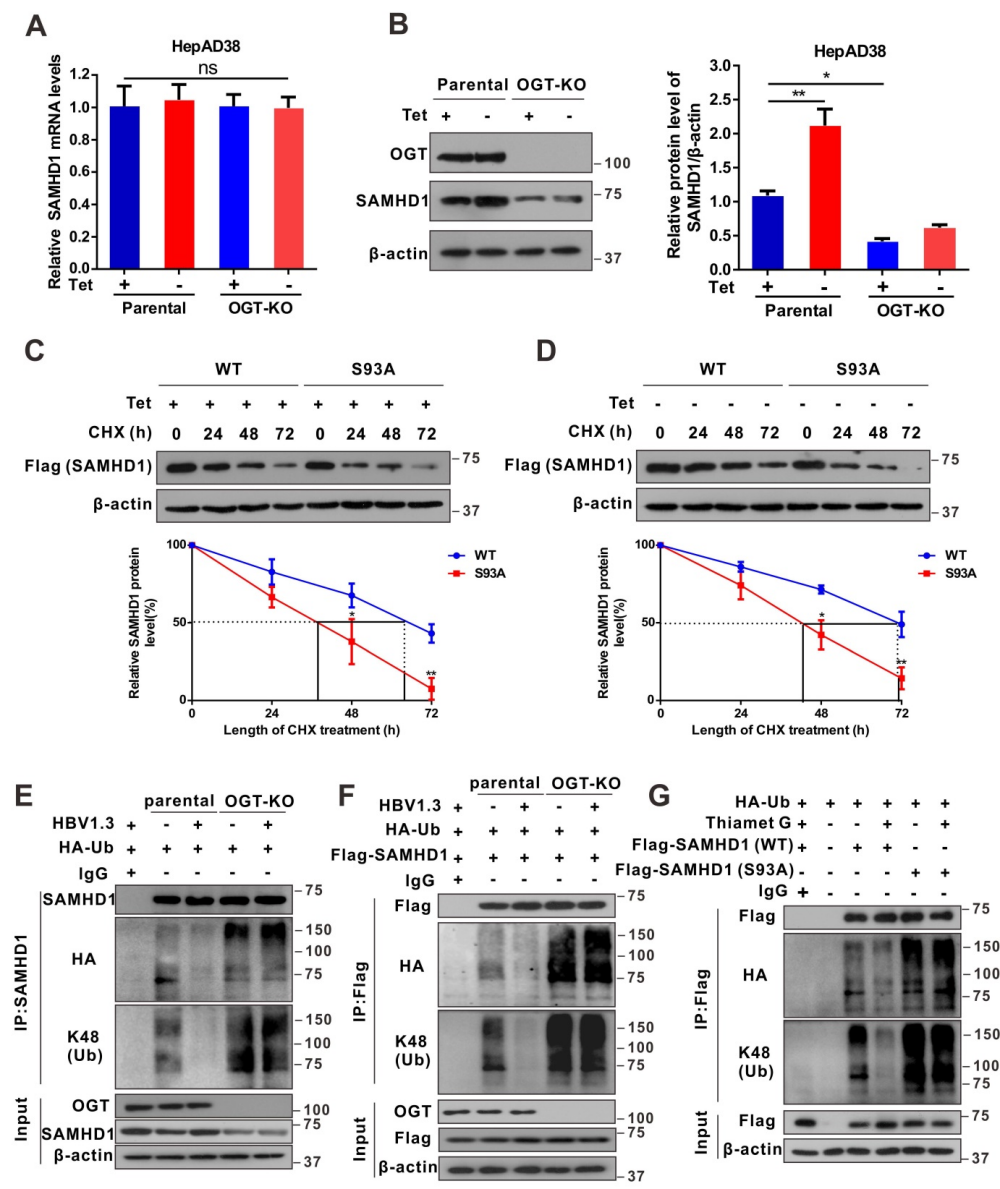
** OGT-mediated O-GlcNAcylation on Ser93 enhances SAMHD1 stability.** (**A**-**B**) RT-qPCR quantification of mRNA (A) and protein levels of SAMHD1 in OGT-KO HepAD38 cells (B) n = 3. KO, knockout. (**C**-**D**) Immunoblots of SAMHD1. SAMHD1-KO HepAD38 cells treated with (Off) or without (On) tetracycline were transfected with Flag-tagged SAMHD1 WT or S93A mutant constructs and treated with 100 μM CHX, n = 3. (**E**) SAMHD1 ubiquitination in OGT-knockout HBV-infected HepG2 cells in the presence of HA-tagged ubiquitin. After cell lysis, SAMHD1 was immunoprecipitated using anti-SAMHD1 antibody and Re-IP of SAMHD1 after boiling. Immunoprecipitated and input proteins were probed with the indicated antibodies. (**F**-**G**) OGT-knockout HepG2 cells were co-transfected with HA-Ub and Flag-SAMHD1 (F); HEK293T cells were transfected with Flag-tagged SAMHD1-WT or S93A mutant and treated with 100 μM TMG for 12 h (G). After cell lysis, SAMHD1 was immunoprecipitated using anti-Flag M2 antibody. Immunoprecipitated and input proteins were probed with the indicated antibodies. Data are expressed as the mean ± SD. *P* values were derived from one-way ANOVA in A and B, two-way ANOVA in C and D (*P < 0.05, **P < 0.01).

To map the O-GlcNAcylation site(s) on SAMHD1, Flag-tagged SAMHD1 was purified from HepG2-HBV1.3 cells and analyzed by MS. As shown in Figure 4K, SAMHD1 was O-GlcNAcylated on Ser93 (S93). Interestingly, SAMHD1 S93 is well conserved among mammalian species (Figure 4L). We then generated site-specific point mutant of *SAMHD1*. Mutation of Ser93 to Ala (S93A) largely reduced the O-GlcNAc signal (Figure 4M- N and S5H). To examine the effect of O-GlcNAcylation on SAMHD1 stability, we first analyzed the endogenous SAMHD1 protein levels in HepAD38 cells with OGT knockdown. There was no significant difference in *SAMHD1* mRNA levels between parental and OGT-KO HepAD38 with or without HBV infection (Figure 5A). However, HBV infection enhanced the protein stability of endogenous SAMHD1. The endogenous SAMHD1 was decreased in OGT-KO cells when compared with parental HepAD38 cells, no matter with or without HBV infection (Figure 5B). When SAMHD1-KO HepAD38 cells were reconstituted with either Flag-tagged WT or S93A mutant SAMHD1, SAMHD1 S93A showed attenuated stability compared with WT SAMHD1 (Figure 5C-D). These data suggest that O-GlcNAcylation of SAMHD1 is important for its stability. We further assessed the effect of OGT on SAMHD1 ubiquitination. Total and K48-linked ubiquitination of SAMHD1 were significantly decreased in HBV-infected HepG2 cells; however, the effect of HBV infection on SAMHD1-Ub was minimal in OGT-KO cells (Figure 5E-F). These results suggest that the HBV-induced reduction of SAMHD1-Ub depends on O-GlcNAcylation of this molecule. Furthermore, the administration of TMG dramatically suppressed total and K48-linked ubiquitination of WT SAMHD1; however, the effect on S93A ubiquitination was minimal, indicating that the S93A mutant was more ubiquitinated than WT SAMHD1 (Figure 5G). These data suggest that O-GlcNAcylation of SAMHD1 at Ser93 stabilizes SAMHD1 by counteracting with ubiquitination.

### O-GlcNAcylation of SAMHD1 on Ser93 enhances its antiviral activity

**Figure 6:**
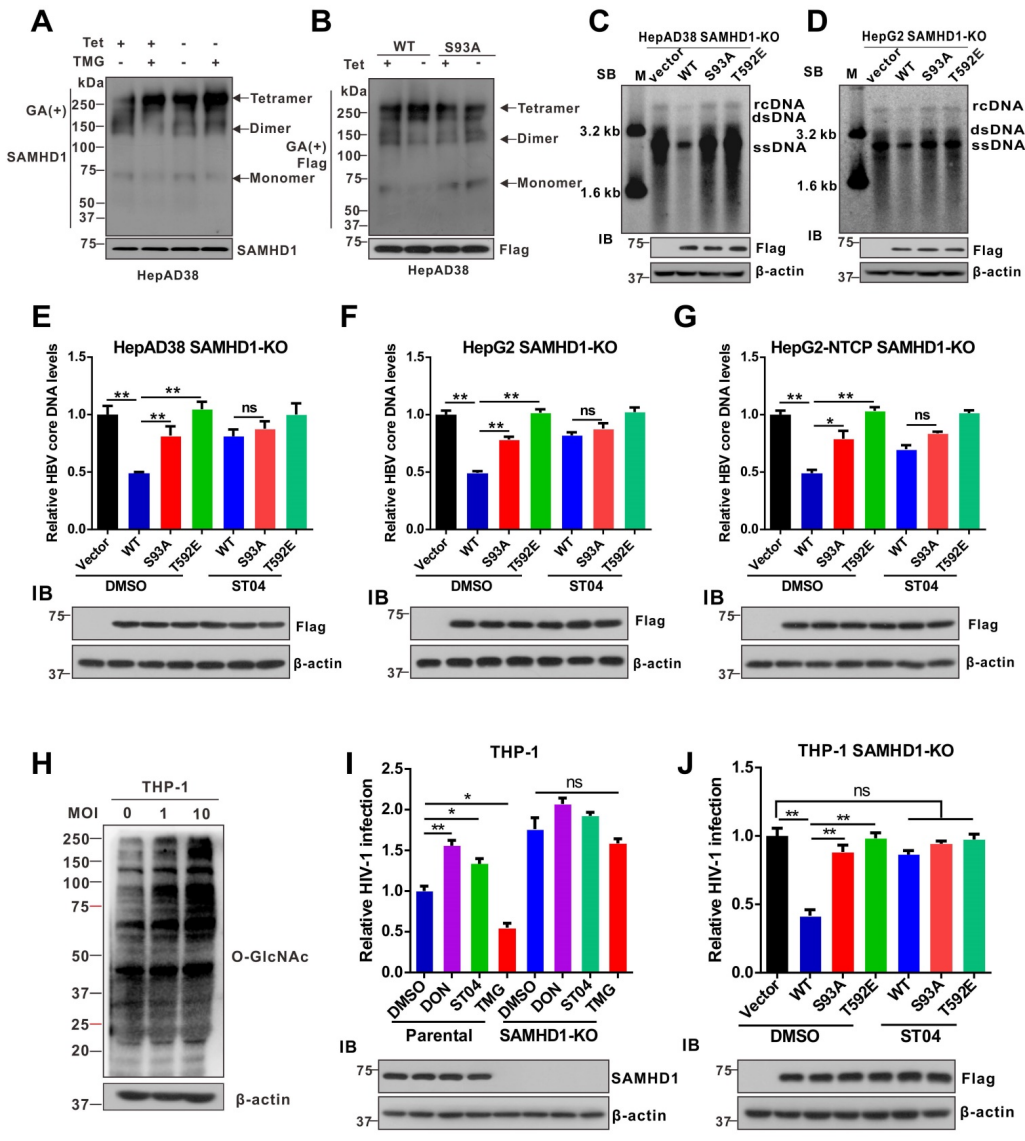
** O-GlcNAcylation of SAMHD1 on Ser93 is important for its antiviral activity. (A)** Analysis of the oligomeric state of SAMHD1 upon HBV infection or under OGA inhibitor TMG treatment. HepAD38 (Tet-off) cells were treated with the Thiamet G. Cells were treated with glutaraldehyde (GA), which was used to crosslink proteins irreversibly, and whole-cell lysates were probed with an anti-SAMHD1 antibody. **(B)** Analysis of the oligomeric state of SAMHD1 WT and S93A mutants upon HBV infection. SAMHD1-KO HepAD38 (Tet-off) cells were transfected with SAMHD1 WT and S93A mutant and treated with glutaraldehyde (GA) and whole-cell lysates were probed with an anti-SAMHD1 antibody. (**C**-**D**) SAMHD1-KO HepAD38 cells with stable HBV-expressing (C) and HBV-infected SAMHD1-KO HepG2 cells (D) were transfected with Flag-tagged SAMHD1-WT, S93A mutant, or T592E mutant constructs. HBV DNA levels were determined by Southern blotting analysis (SB, Southern blot; IB, immunoblot). (**E**-**G**) SAMHD1-KO HepAD38 cells with stable HBV-expressing (E), HBV-infected SAMHD1-KO HepG2 (F) and SAMHD1-KO HepG2-NTCP cells (G) were transfected with the above plasmids described in (C). HBV core DNA levels were determined by qPCR. n = 3 for biological repeats. (**H**) SAMHD1 KO-THP-1 cells were differentiated overnight with phorbol myristate acetate (PMA, 100 μM) before infecting with HIV-1-LUC-G (MOI = 0, 1, or 10) for 48 h. Thereafter, the cells were lysed and total O-GlcNAc levels were determined via western blotting. β-actin was used as a loading control. (**I**) SAMHD1-WT and -KO THP-1 cells were differentiated overnight and infected with HIV-1-LUC-G (MOI = 1) for 24 h. Cells were then treated with DON (30 μM, 24 h), ST04 (100 μM, 24 h), or TMG (100 μM, 48 h), and luciferase activity was measured. n = 3. (**J**) SAMHD1-KO THP-1 cells were differentiated overnight with PMA and transfected with Flag-tagged SAMHD1-WT, S93A mutant, or T592E mutant plasmids for 48 h, then infected with HIV-1-LUC-G (MOI = 1) for another 24 h until cell lysis. Luciferase activity was measured and normalized for protein concentration. n = 3. Data are expressed as the mean ± SD. *P* values were derived from one-way ANOVA in E-G, I-J. (* *P* < 0.05, ** *P* < 0.01). DON, 6-diazo-5-oxo-L-norleucine; ST04, ST045849; TMG, Thiamet G.

It is known that the tetramer conformation of SAMHD1 is required for its dNTPase activity 23. Herein, we sought to determine whether O-GlcNAcylation of SAMHD1 affects its tetramerization and dNTPase activity. Recombinant WT and S93A SAMHD1 were expressed and purified (Figures S6A and S6B). We found that HBV infection or Thiamet G (TMG, an OGA inhibitor) enhanced SAMHD1 tetramerization in HepAD38 cells (Figure 6A). However, the S93A mutation reduced tetramerization (Figure 6B) and dNTPase activity *in vitro* (Figure S6C-D). To test the effect of S93 O-GlcNAcylation on SAMHD1 antiviral activity, we deleted endogenous SAMHD1 in our HBV cell models and THP-1 cells using CRISPR-Cas9-mediated gene editing and transfected WT or SAMHD1 variants into SAMHD1-knockout HepAD38 (Tet-off), AdHBV-1.3-infected HepG2, and HepG2-NTCP cells. A phospho-mimetic mutation (T592E) was used as a control that also decreased SAMHD1 dNTPase activity and abrogated its antiviral activity 40. Both Southern blotting (Figure 6C-D) and qPCR (Figure 6E-G) results indicated that S93A mutation impairs the ability of SAMHD1 to inhibit HBV replication *in vitro*. A previous study showed that SAMHD1 dNTPase activity is essential for HIV-1 restriction 21,41. Therefore, we investigated the effect of SAMHD1 O-GlcNAcylation on HIV-1 infection. THP-1 cells were infected with a VSV-G protein-pseudotyped HIV-1 molecular clone carrying the luciferase gene reporter, and virus replication was assessed by quantifying luciferase activity. Our results showed that protein O-GlcNAcylation was increased upon HIV-1 infection in THP-1 cells (Figure 6H). Meanwhile, reduced O-GlcNAcylation by DON and ST04 treatment significantly promoted HIV-1 infection, whereas hyper-O-GlcNAcylation through TMG treatment suppressed HIV-1 infection in SAMHD1-expressing THP-1 cells; however, these effects were minimal in SAMHD1-KO THP-1 cells (Figure 6I). Further, WT or SAMHD1 variants were transfected into SAMHD1-KO THP-1 cells. The S93A mutation significantly impaired the ability of SAMHD1 to restrict HIV-1 replication in this single-round HIV-1 infection model. However, neither SAMHD1 WT nor S93A could markedly restricts the HIV-1 replication in presence of the OGT inhibitor ST04 (Figure 6J). Taken together, these results indicate that O-GlcNAcylation of SAMHD1 S93 promotes its antiviral activity *in vitro*.

### HBV infection promotes UDP-GlcNAc biosynthesis and O-GlcNAcylation *in vivo*

**Figure 7:**
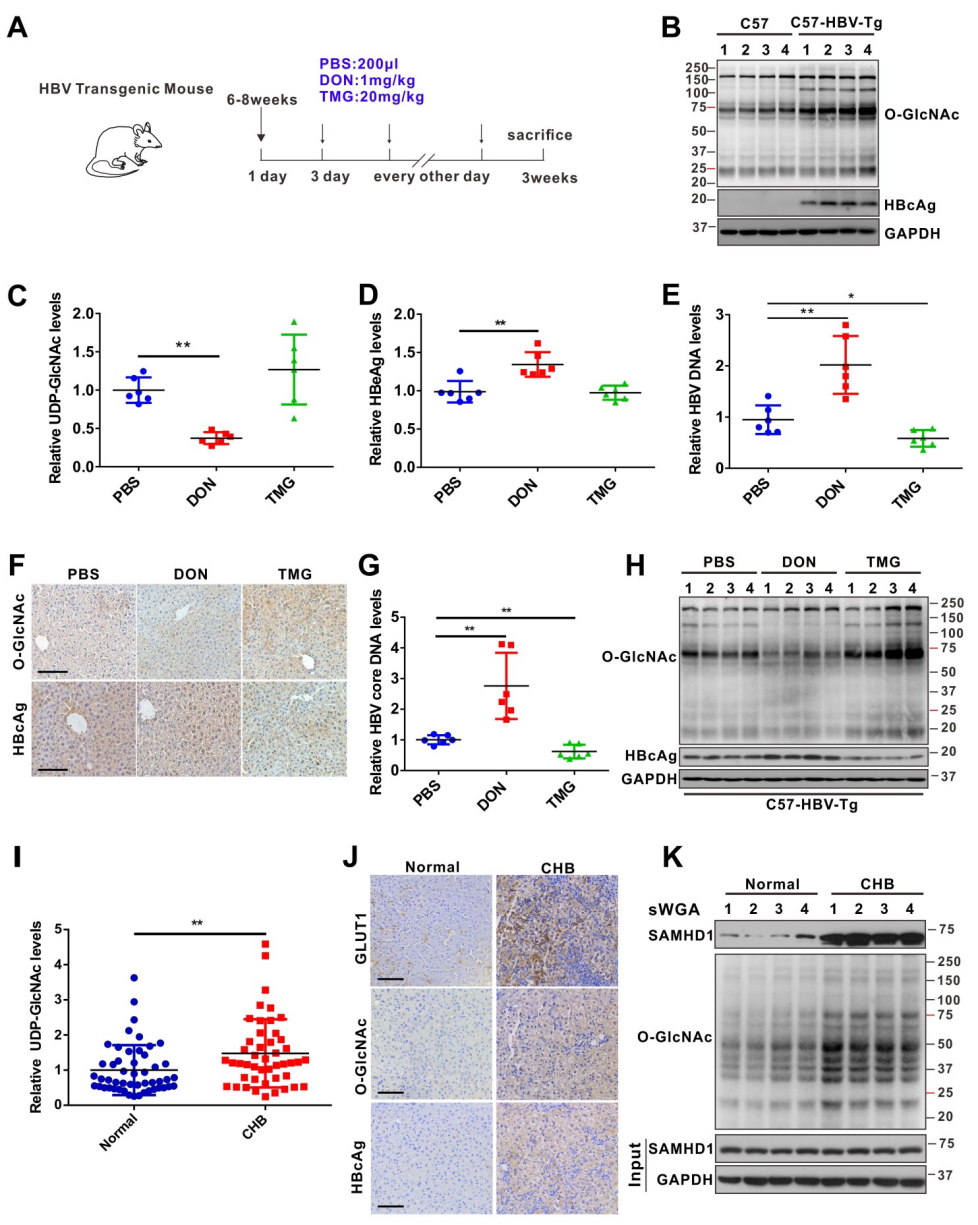
** HBV infection promotes UDP-GlcNAc biosynthesis and protein O-GlcNAcylation *in vivo.***(**A**) Six- to eight-week-old HBV transgenic mice were intraperitoneally injected with DON (1 mg/kg body weight), TMG (20 mg/kg body weight) or PBS (control) every other day for 10 times. The mice were sacrificed on day 20 post-treatment. (**B**) Immunoblotting of total O-GlcNAc in HBV transgenic mice. (**C**) Fold change of UDP-GlcNAc in mouse liver tissues was determined by UHPLC-QTOF-MS. n = 6 per group. (**D**-**E**) Serum HBeAg and HBV DNA levels in mice. n = 6 per group. (**F**) O-GlcNAc and HBcAg detection in mouse liver tissues. Scale bar, 50 μm. (**G**) Quantification of HBV core DNA levels in mouse liver tissues using qPCR. n = 6. (**H**) Immunoblot of total O-GlcNAc in HBV transgenic mice treated as in (A). (**I**) Relative change of UDP-GlcNAc in the liver tissues of patients with CHB was determined by UHPLC-QTOF-MS (Normal = 50, CHB = 46). (**J**) GLUT1, O-GlcNAc, and HBcAg detection in liver tissue specimens from patients with CHB. Scale bar, 50 μm. (**K**) Liver tissue lysates from patients with CHB were purified using sWGA-conjugated agarose beads and probed with an anti-SAMHD1 or anti-O-GlcNAc antibody. Data are expressed as the mean ± SD. *P* values were derived from one-way ANOVA in C-E, G, from Mann-Whitney U test in I (* *P* < 0.05, ** *P* < 0.01).

We used an HBV-transgenic (HBV-Tg) mouse model to verify our results *in vivo* (Figure 7A).The level of O-GlcNAcylation was significantly higher in the liver tissues of HBV-Tg mice than in those of normal C57BL/6 mice (Figure 7B). Consistent with our *in vitro* data, the administration of DON significantly reduced UDP-GlcNAc levels (Figure 7C) and stimulated HBV replication (Figure 7D-F) in HBV infected mouse model, whereas the administration of OGA inhibitor TMG decreased serum HBV DNA (Figure 7E), liver HBcAg (Figure 7F) and HBV DNA (Figure 7G) levels in mice. Protein O-GlcNAcylation levels in the liver tissues of HBV-Tg mice were increased upon TMG administration, but decreased upon DON administration (Figure 7H). These results indicate that TMG may suppress HBV replication by increasing host protein O-GlcNAcylation *in vivo*.

Finally, we examined UDP-GlcNAc biosynthesis and O-GlcNAcylation levels in patients with chronic hepatitis B (CHB). The levels of serum UDP-GlcNAc (Figure 7I), GLUT1 protein (Figure 7J), and total O-GlcNAcylation (Figure 7J-K) were markedly higher in the liver tissues of patients with CHB than in those of normal controls. In addition, endogenous SAMHD1 O-GlcNAcylation was significantly increased in the liver tissues of the patients with CHB (Figure 7K). Overall, our study suggests that HBV infection upregulates GLUT1 expression and increases UDP-GlcNAc biosynthesis and O-GlcNAcylation *in vivo*. As an essential O-GlcNAcylated protein, SAMHD1 can exert its antiviral activity and elicit a robust host innate immune response against HBV infection.

## Discussion

Although previous studies have demonstrated that HBV infection can alter glucose metabolism in host cells, the role and underlying mechanisms of metabolic regulation of antiviral immune responses remain elusive. In this study, we demonstrate that HBV increases GLUT1 expression on the hepatocyte surface, thereby facilitating glucose uptake. This enhanced nutrient state consequently provides substrates to HBP to produce UDP-GlcNAc, leading to an increase in protein O-GlcNAcylation. Importantly, we found that pharmacological or transcriptional inhibition of HBP and O-GlcNAcylation can promote HBV replication. Furthermore, we showed that OGT-mediated O-GlcNAcylation of SAMHD1 on Ser93 is critical for its antiviral activity. Our results therefore indicate that SAMHD1 O-GlcNAcylation plays a positive role in host antiviral innate immunity against HBV.

Similar to the metabolic reprogramming in proliferating cancer cells, viruses reprogram host cell metabolism. It has been reported that several viruses increase glucose consumption and reprogram glucose metabolism in the host cell 42,43. GLUT1 expression was increased in host cells infected with HIV-1 44,45, Kaposi's sarcoma-associated herpes virus 46, dengue virus 47, and Epstein-Barr virus 48. As the main glucose transporter, GLUT1 has been shown to be directly associated with gp46, a cleavage product of the envelope glycoprotein of human T cell leukemia virus 1 (HTLV-1). GLUT1 serves as its receptor and is involved in efficient entry of HTLV 49,50. The GLUT1-mediated metabolic pathway is essential for HIV-1 infection in human CD4^+^ T cells and thymocytes 44,51,52. Moreover, HBV infection upregulates the expression level of *GLUT1* to facilitate glucose uptake and meet the cell's energetic needs in response to viral infection 53. Our findings are consistent with previous transcriptome-wide analyses, which have also shown HBV-mediated upregulation of GLUT1 37. It has been suggested that the HBV pre-S2 mutant increases *GLUT1* expression via mammalian target of rapamycin (mTOR) signaling cascade, leading to enhanced glucose uptake 54. However, the precise molecular mechanism by which HBV upregulates GLUT1 remains further investigation.

The enhanced glucose uptake by glucose transporters not only accelerates glycolysis, but may also increase flux into branch pathways, such as the pentose phosphate pathway and HBP, which occur in cancer cells 55. Previous studies have reported that HBP plays an important role in host innate immunity. Consistent with the results of a previous study with HepG2.2.15 cells 35, our results showed that HBV infection can promote HBP activity and increase UDP-GlcNAc levels in different cell models. Li *et al.* reported that enhanced HBP activity is essential for HBV replication because pharmacological or transcriptional suppression of *GFPT1* inhibits HBV replication in HepG2.2.15 cells. However, they did not use an *in vivo* HBV model to study the underlying mechanism. In contrast, we showed that blockade of HBP promotes HBV replication, whereas stimulation of HBP significantly suppresses HBV replication both *in vitro* and *in vivo*. In addition, we observed similar results upon HIV-1 infection using a single-round infection model. Although we could not exclude the possibility that differences between HBV cell models cause this discrepancy, our results show that increased HBP flux and hyper-O-GlcNAcylation can upregulate host antiviral innate response. Several other studies have reported that HBP and/or protein O-GlcNAcylation promotes host antiviral immunity against RNA viruses, including VSV 13, influenza virus 14, and hepatitis C virus 15. Thus, the present study confirms and expands our current understanding of the antiviral activity of HBP and protein O-GlcNAcylation upon DNA virus infection, which is similar to its antiviral activity upon infection by certain RNA viruses.

By characterizing the role of protein O-GlcNAcylation during HBV replication, we uncovered SAMHD1 as an important target of OGT and established a link between O-GlcNAcylation and antiviral immune response against HBV infection. SAMHD1, an effector of innate immunity, can restrict most retroviruses (such as HIV-1) and several DNA viruses (including HBV) by depleting the intracellular pool of dNTPs 39. Herein, we identified Ser93 as a key O-GlcNAcylation site on SAMHD1 using LC-MS/MS. Importantly, loss of O-GlcNAcylation by S93A mutation increased K48-linked ubiquitination, thus decreasing the stability and dNTPase activity of SAMHD1, suggesting that O-GlcNAcylation promotes the antiviral activity of SAMHD1. Interestingly, S93A mutation did not completely destroy O-GlcNAcylation of SAMHD1, indicating that there may be other O-GlcNAcylation sites, a topic which needs further investigation.

Many of the previous studies have demonstrated that several DNA and RNA virus infections can regulate the stability of SAMHD1. HCMV infection promotes degradation of SAMHD1 at late stages through the Cullin-RING-E3 ligase complexes 56. Enterovirus 71 infection increases TRIM21 (an E3 ubiquitin ligase) expression, and TRIM21 directly interacts and degrades SAMHD1 through the proteasomal pathway 25. On the contrary, HBV-encoded proteins such as hepatitis B virus-X (HBx) benefits virus replication by directly or indirectly degrading multiple cellular restriction factors. SELENBP1 is markedly decreased in HBx-expressing cells 57. HBx has been found to hijack the cellular DDB1-containing E3 ubiquitin ligase to target Smc5/6 for degradation 58. Our data showes that HBV infection decreases the ubiquitination and enhances the stability of SAMHD1, processes which are mediated by enhanced SAMHD1 O-GlcNAcylation; however, whether HBV proteins directly or indirectly promotes the stability of SAMHD1 needs to be verified in further studies.

Because our results demonstrated the importance of protein O-GlcNAcylation in host antiviral innate immunity against HBV, we proposed that an increase in SAMHD1 O-GlcNAcylation by inhibiting OGA activity could be used as a potential antiviral strategy. This is in line with recent results indicating that increased MAVS O-GlcNAcylation is essential to activate host innate immunity against RNA viruses 13,14. However, hyper-O-GlcNAcylation has been reported to stabilize several oncogenic factors in several cancers associated with oncogenic virus infection 59. Human papilloma virus 16 E6 protein can upregulate OGT and stabilize c-MYC via O-GlcNAcylation, thus promoting HPV-induced carcinogenesis 60. Herzog *et al*. demonstrated that protein O-GlcNAcylation is involved in HCV-induced disease progression and carcinogenesis 15. Thus, the role of enhanced protein O-GlcNAcylation in HBV pathogenesis and the antiviral response remains to be further studied.

In conclusion, we uncovered a link between metabolic reprogramming and antiviral innate immunity against HBV infection. We demonstrated that HBV infection upregulates GLUT1 expression and promotes HBP flux *in vitro* and *in vivo*. In addition, increased UDP-GlcNAc biosynthesis and hyper-O-GlcNAcylation can enhance host antiviral innate response. Mechanistically, OGT-mediated O-GlcNAcylation of SAMHD1 on Ser93 stabilizes SAMHD1 and enhances its antiviral activity. This study broadens our understanding of SAMHD1 post-translational modification and provides new insights into the importance of HBP and protein O-GlcNAcylation in antiviral innate immunity.

## Supplementary Material

Supplementary figures and tables 1-2.

Supplementary table 3.

## Abbreviations

CHB: chronic hepatitis B
dNTPase: dNTP triphosphohydrolase
DON: 6-diazo-5-oxo-L-norleucine
GlcNAc: N-acetylglucosamine
GLUT1: glucose transporter 1
HBP: hexosamine biosynthesis pathway
HBV: hepatitis B virus
HBx: hepatitis B virus-X
HCMV: human cytomegalovirus
HIV-1: human immunodeficiency virus type 1
HSV-1: herpes simplex virus-1
NTCP: solute carrier family 10 member 1
OGA: O-GlcNAcase
O-GlcNAc: O-linked β-N-acetylglucosamine
OGT: O-GlcNAc transferase
RIPK3: receptor-interacting serine/threonine-protein kinase 3
SAMHD1: sterile alpha motif and histidine/aspartic acid domain-containing protein 1
ST04: ST045849
STAT3: signal transducer and activator of transcription-3
TMG: Thiamet G
UDP-GlcNAc: N-acetylglucosamine
UTP: uridine triphosphate
VSV-G: vesicular stomatitis virus G
