# Supplementary material for: Hexosamine biosynthetic pathway promotes the antiviral activity of SAMHD1 by enhancing O-GlcNAc transferase-mediated protein O-GlcNAcylation
Source: Theranostics. 2021 Jan 1;11(2):805–23. doi: 10.7150/thno.50230 (PMC7738853; doi:10.7150/thno.50230)
Supplement: Supplementary file 1 — Supplementary figures and tables 1-2. [file thnov11p0805s1.pdf]

Hexosamine biosynthetic pathway promotes the antiviral activity of SAMHD1 by enhancing O-GlcNAc transferase-mediated protein O-GlcNAcylation

Jie Hu[1,#], Qingzhu Gao[1,#], Yang Yang[1,#], Jie Xia[1,#], Wanjun Zhang[2], Yao Chen[1], Zhi Zhou[1], Lei Chang[2], Yuan Hu[1], Hui Zhou[3], Li Liang[1],Xiaosong Li[4], Quanxin Long[1], Kai Wang[1, *], Ailong Huang[1, *], Ni Tang[1, *]

Supplementary Figure Legends

Figure S1. HBV infection enhances HBP activity and upregulates GLUT1 expression. (**A**) KEGG pathway enrichment analysis of metabolites detected using metabolomics assay in HepG2 cells infected with AdHBV1.3 or AdGFP for 72 h. (**B-C**) Fold change in UDP-GlcNAc (B) and glucose (C) levels in HBV1.3-infected HepG2 cells was determined using LC-MS/MS targeted metabolomics assay, n = 6. (**D**) Immunoblot of total O-GlcNAc from PHH cells treated for the indicated periods. (**E-F**) RT-qPCR quantification (E) and immunofluorescence staining (F) of GLUT1 in HepG2 cells, DAPI (blue) was used to counterstain nuclei; n = 3. Scale bar, 10 μm. (**G-H**) Immunoblots of OGA, OGT, p-GFPT1, and GFPT1 from HepG2, HepAD38 (G), and HepG2-NTCP (H) cells. Data are expressed as the mean ± SD. *P* values were derived from unpaired, two-tailed Student's *t*-test in B-C and E (***P* < 0.01).

Figure S2. Role of O-GlcNAc modification in regulating HBV replication in HepG2 cells. (**A-C**) Immunoblot of total O-GlcNAc from HBV1.3-infected HepG2 cells treated with or without GLUT1 inhibitor WZB117 (50 μM) (A), GFPT1 inhibitor DON (30 μM) (B), or OGT inhibitor ST04 (100 μM) (C) for 72 h. (**D-F**) Quantification of HBV core DNA levels in HBV1.3-infected HepG2 cells treated as indicated using qPCR, n = 3. (**G**) Immunoblot of total O-GlcNAc from HBV1.3-infected HepG2 cells treated with or without OGA inhibitor TMG (100 μM) for 72 h. (**H**) Quantification of HBV core DNA levels in

HBV1.3-infected HepG2 cells treated as in (G) using qPCR, n = 3. Data are expressed as the mean ± SD. *P* values were derived from unpaired, two-tailed Student's *t*-test in D-F and H; (***P* < 0.01, ****P* < 0.001).

Figure S3. O-GlcNAc modification regulates HBV replication in HepG2-NTCP cells and PHH cells. (**A-E**) Immunoblot of total O-GlcNAc from HBV-infected cells treated as indicated. (**F-J**) Quantification of HBV core DNA levels in HBV-infected cells treated as indicated using qPCR, n = 3. (**K-L**) Immunoblot of total O-GlcNAc from HBV-infected cells treated with or without TMG (100 μM) for 72 h. (**M-N**) qPCR quantification of HBV core DNA levels in HBV-infected cells treated with or without TMG, n = 3. Data are expressed as the mean ± SD. *P* values were derived from unpaired, two-tailed Student's *t*-test in F-J and M-N (***P* < 0.01, ****P* < 0.001).

Figure S4. Inhibition of protein O-GlcNAcylation enhances HBV replication. (**A-B**) Immunoblot of total O-GlcNAc from HBV1.3-infected HepG2 cells following shRNA-mediated GFPT1 or OGT knockdown. (**C-D**) Quantification of HBV core DNA levels in HBV1.3-infected HepG2 cells treated as above using qPCR, n = 3. (**E**) Immunoblot of total O-GlcNAc from HBV1.3-infected OGA knockdown HepG2 cells. (**F**) Quantification of HBV core DNA levels in HBV1.3-infected HepG2 cells treated as in (**E**) using qPCR, n = 3. Data are expressed as the mean ± SD. *P* values were derived from one-way

ANOVA in C-D and F; (***P* < 0.01, ****P* < 0.001).

Figure S5. HBV infection promotes SAMHD1 O-GlcNAcylation. (**A**) IP-LC-MS/MS analysis of O-GlcNAc modified proteins. HepAD38 cell lysates were immunoprecipitated using an anti-O-GlcNAc antibody. (**B**) HEK293T cells, (**C**) Tetracycline-inducible HepAD38 cells, (**D**) HepG2-NTCP cells were transfected with the Flag-SAMHD1 construct or the control vector for 48 h, and treated with 100 μM TMG for 12 h (B), Cells lysates were purified using sWGA-conjugated agarose beads and probed with an anti-Flag or anti-O-GlcNAc antibody. (**E**) PHH cells were infected with HBV for 72 h. After cell lysis, O-GlcNAc-modified proteins were purified using the sWGA-conjugated agarose beads and probed with an anti-SAMHD1 antibody. The immunoprecipitated and input proteins were probed with an anti-O-GlcNAc or anti-SAMHD1 antibody. (**F-G**) Southern blot assay in stable HBV-expressing HepAD38 and SAMHD1-KO HepAD38 cells. rc DNA, relaxed circular DNA; ds DNA, double-stranded DNA; ss DNA, single-stranded DNA. (**H**) SAMHD1-KO HepG2-NTCP cells were transfected with either empty vector, Flag-tagged SAMHD1 WT or the S93A mutant, and treated with 100 μM PUGNAc for 12 h. Cells lysates were purified using sWGA-conjugated agarose beads and probed with an anti-Flag or anti-O-GlcNAc antibody.

Figure S6. SAMHD1 protein expression, purification, and dNTPase

activity. (**A-B**) His-tagged pET28a-SAMHD1 constructs were expressed in *Escherichia coli* BL21 (DE3) cells and induced with 0.2 mM IPTG. Cell lysates were purified using Ni-NTA affinity column. The purified target protein was stained with Coomassie Brilliant Blue dye. Lane 1, molecular-mass markers; Lane 2, cell supernatant after ultrasonic crash; Lane 3, Ni–NTA affinity column flow through fraction; Lane 4-6, eluted target protein with 20 to 400 mM imidazole; Lane 7, eluted protein following hyperfiltration. (**C-D**) HPLC assay to determine SAMHD1 dNTPase activity. SAMHD1-WT (C) and SAMHD1-S93A (D) constructs were purified using Ni-NTA affinity and size-exclusion chromatography. Nucleotide hydrolysis reactions were performed in SAMHD1 buffer containing 500 μM dNTPs and 500 μM SAMHD1-WT, the peak height in Y-axis(mAU) refers to the maximum concentration of ‘dG’, X-axis(min) refers to retention time. The reactions were terminated using 10 mM EDTA. Protein was separated using an Amicon Ultra 0.5-mL 10-kDa filter and analyzed using a Venusil MP-C18 column on an HPLC system.

Figure S1

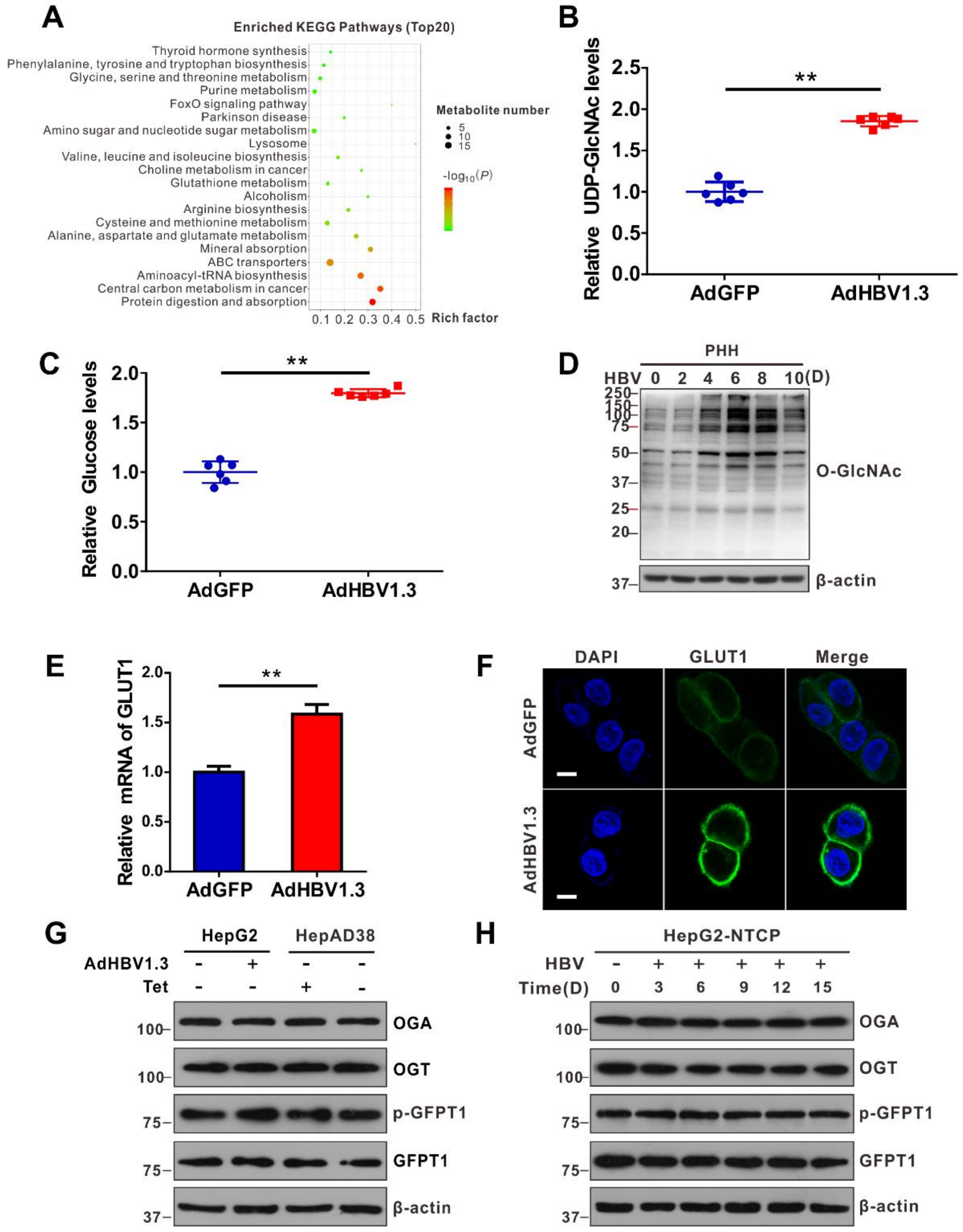
A
Enriched KEGG Pathways (Top20)
Thyroid hormone synthesis
Phenylalanine, tyrosine and tryptophan biosynthesis
Glycine, serine and threonine metabolism
Purine metabolism
FoxO signaling pathway
Parkinson disease
Amino sugar and nucleotide sugar metabolism
Lysosome
Valine, leucine and isoleucine biosynthesis
Choline metabolism in cancer
Glutathione metabolism
Alcoholism
Arginine biosynthesis
Cysteine and methionine metabolism
Alanine, aspartate and glutamate metabolism
Mineral absorption
ABC transporters
Aminoacyl-tRNA biosynthesis
Central carbon metabolism in cancer
Protein digestion and absorption
0.1 0.2 0.3 0.4 0.5 Rich factor
Metabolite number
5
10
15
-log10(P)
B
Relative UDP-GlcNAc levels
2.5
2.0
1.5
1.0
0.5
0.0
**
AdGFP
AdHBV1.3
C
Relative Glucose levels
2.0
1.5
1.0
0.5
0.0
**
AdGFP
AdHBV1.3
D
PHH
HBV 0 2 4 6 8 10(D)
250
150
100
75
50
37
25
20
O-GlcNAc
37
β-actin
E
Relative mRNA of GLUT1
2.0
1.5
1.0
0.5
0.0
**
AdGFP
AdHBV1.3
F
DAPI
GLUT1
Merge
AdGFP
AdHBV1.3
G
HepG2
HepAD38
AdHBV1.3 - + - -
Tet - - + -
100 OGA
100 OGT
75 p-GFPT1
75 GFPT1
37 β-actin
H
HepG2-NTCP
HBV - + + + + +
Time(D) 0 3 6 9 12 15
100 OGA
100 OGT
75 p-GFPT1
75 GFPT1
37 β-actin

Figure S2

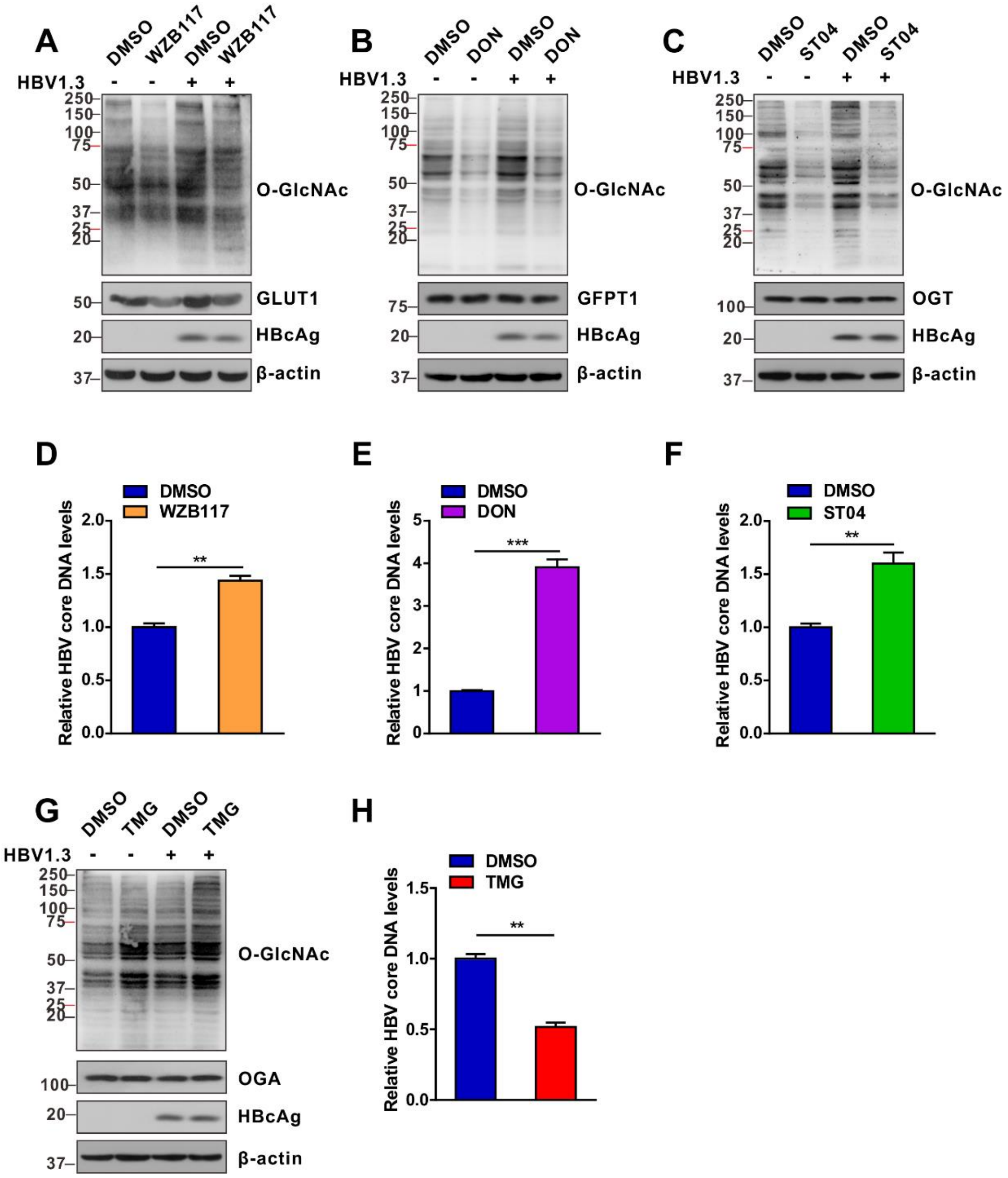

A
DMSO
WZB117
DMSO
WZB117
HBV1.3
O-GlcNAc
GLUT1
HBcAg
β-actin
B
DMSO
DON
GFPT1
C
ST04
OGT
D
Relative HBV core DNA levels
E
F
G
TMG
OGA
H

Figure S3

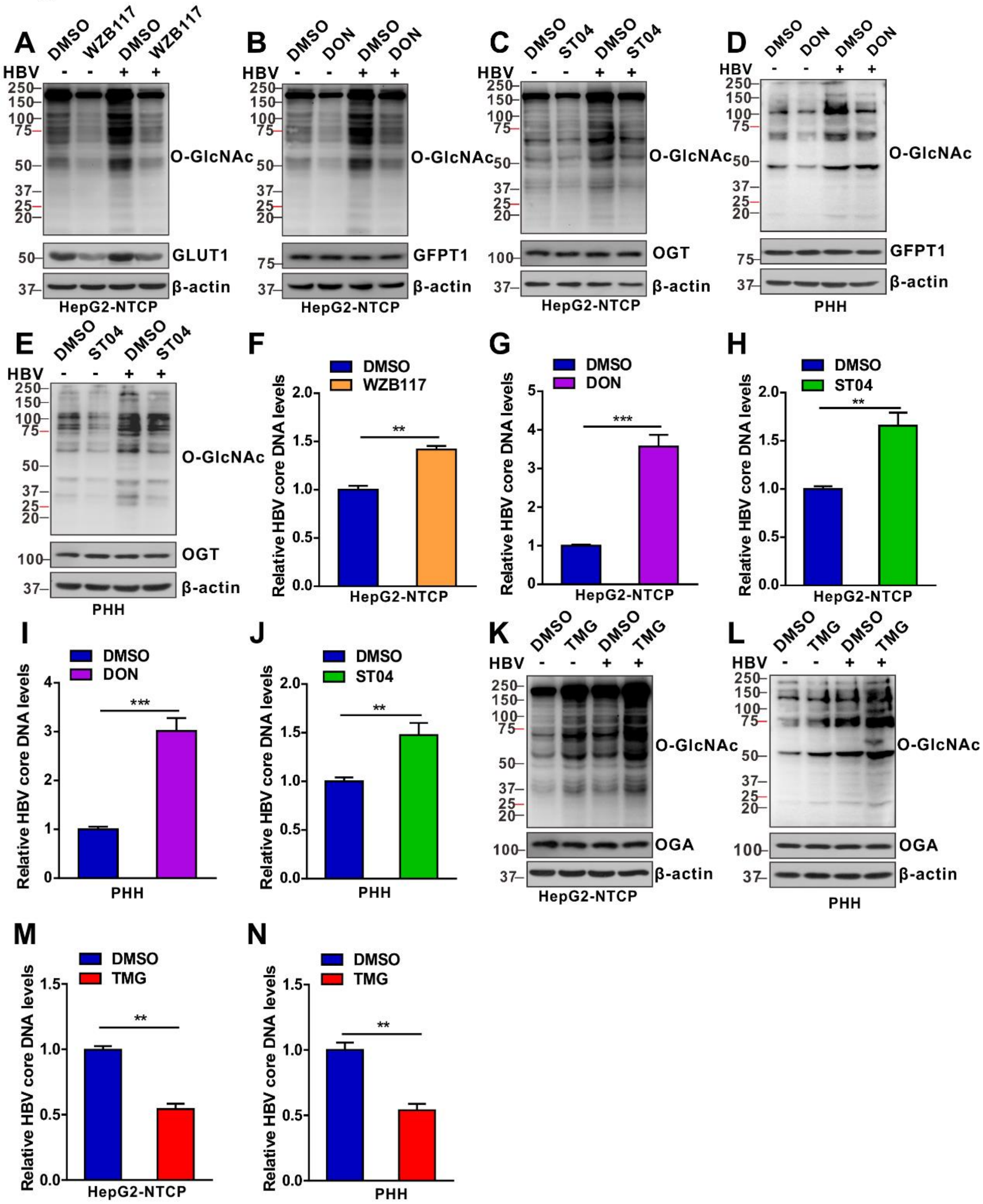
A
B
C
D
E
F
G
H
I
J
K
L
M
N
HBV
DMSO
WZB117
DON
ST04
TMG
O-GlcNAc
GLUT1
GFPT1
OGT
OGA
β-actin
HepG2-NTCP
PHH
Relative HBV core DNA levels
250
150
100
75
50
37
25
20
**
***

Figure S4

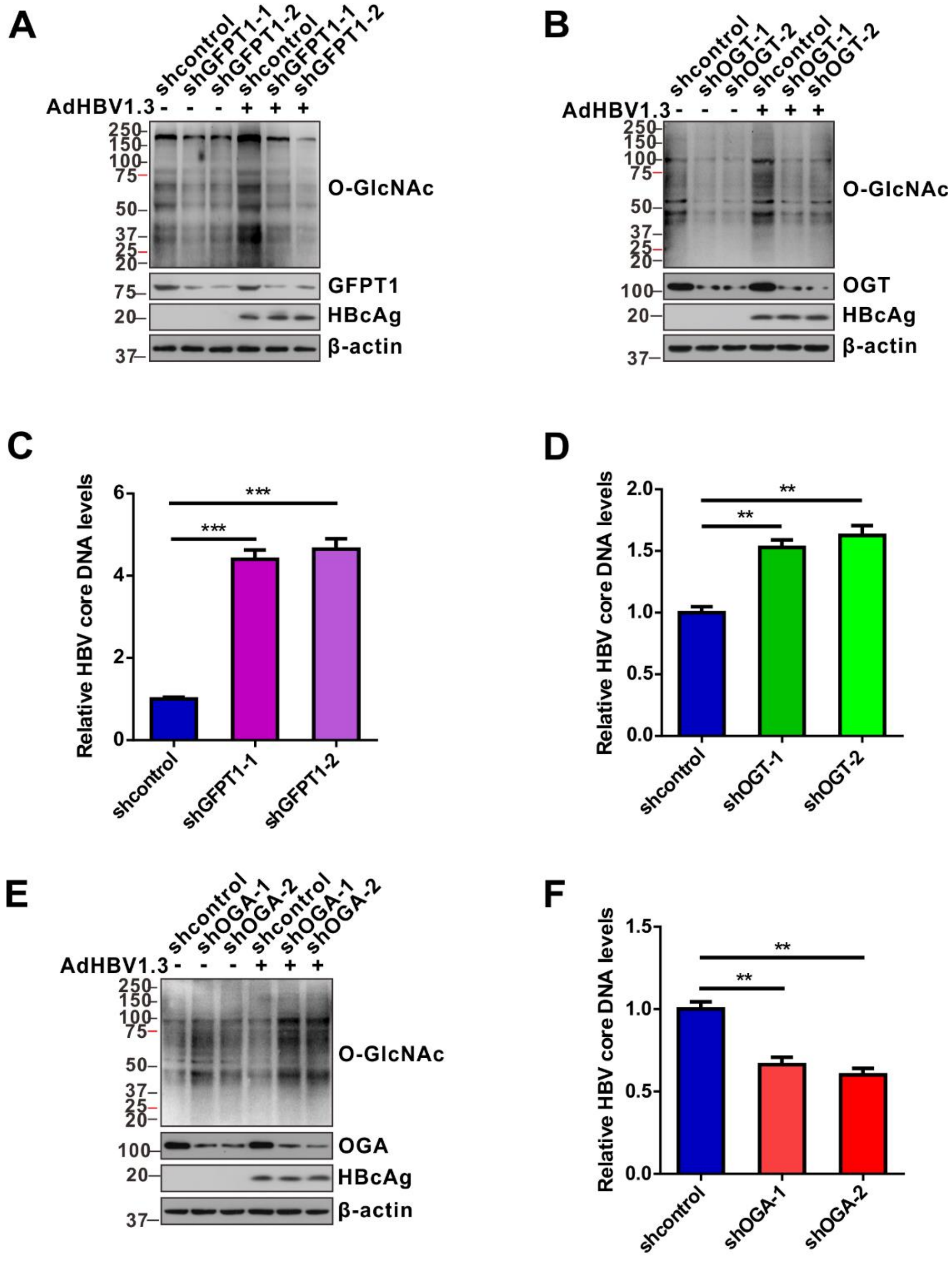
A
shcontrol
shGFPT1-1
shGFPT1-2
shcontrol
shGFPT1-1
shGFPT1-2
AdHBV1.3 - - - + + +
250
150
100
75
50
37
25
20
O-GlcNAc
GFPT1
HBcAg
β-actin
B
shcontrol
shOGT-1
shOGT-2
shcontrol
shOGT-1
shOGT-2
AdHBV1.3 - - - + + +
O-GlcNAc
OGT
HBcAg
β-actin
C
Relative HBV core DNA levels
***
***
shcontrol
shGFPT1-1
shGFPT1-2
D
Relative HBV core DNA levels
**
**
shcontrol
shOGT-1
shOGT-2
E
shcontrol
shOGA-1
shOGA-2
shcontrol
shOGA-1
shOGA-2
AdHBV1.3 - - - + + +
O-GlcNAc
OGA
HBcAg
β-actin
F
Relative HBV core DNA levels
**
**
shcontrol
shOGA-1
shOGA-2

Figure S5

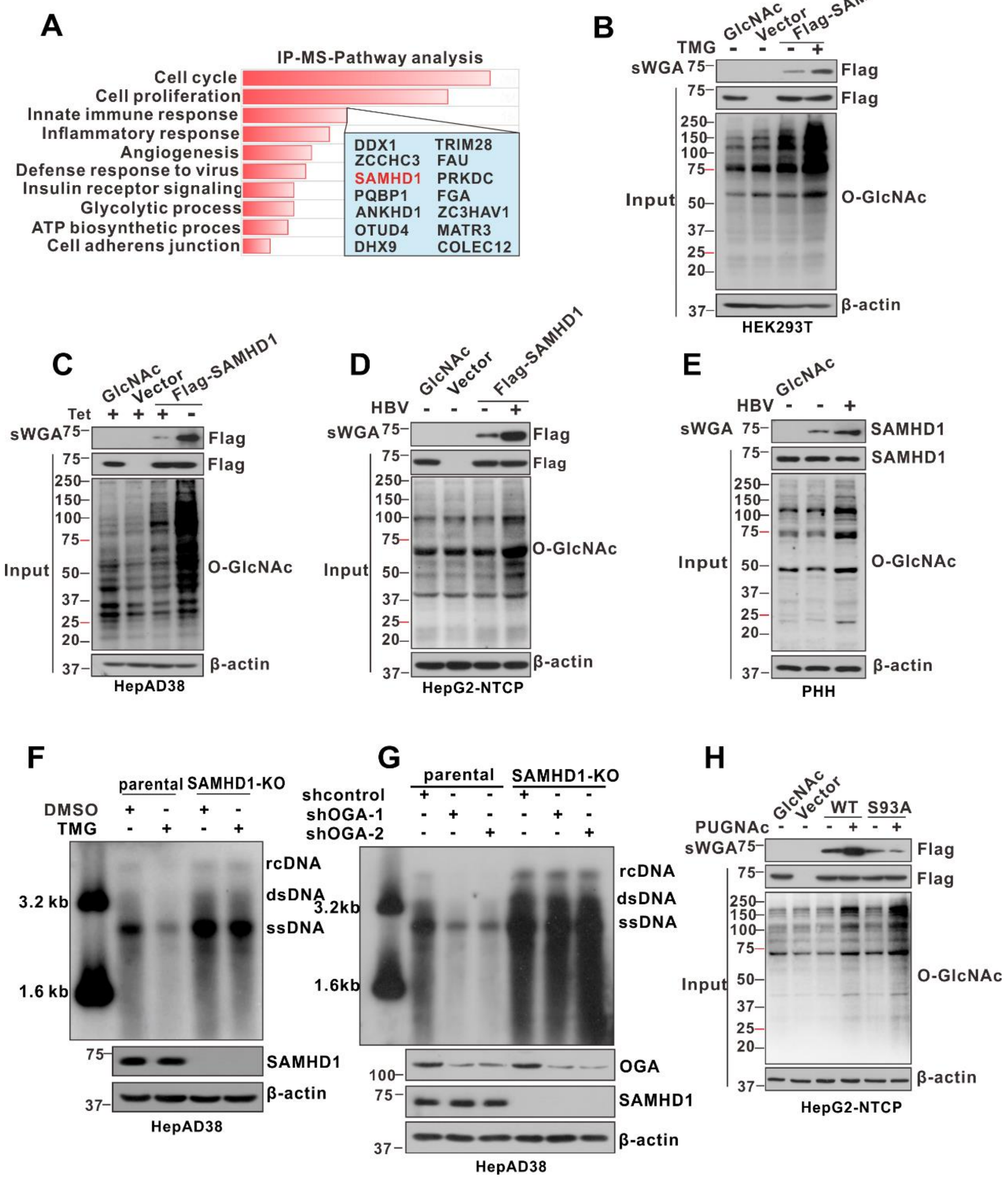
A
IP-MS-Pathway analysis
Cell cycle
Cell proliferation
Innate immune response
Inflammatory response
Angiogenesis
Defense response to virus
Insulin receptor signaling
Glycolytic process
ATP biosynthetic proces
Cell adherens junction
DDX1 TRIM28
ZCCHC3 FAU
SAMHD1 PRKDC
PQBP1 FGA
ANKHD1 ZC3HAV1
OTUD4 MATR3
DHX9 COLEC12
B
GlcNAc
Vector
Flag-SAMHD1
TMG
sWGA
Flag
Input
O-GlcNAc
β-actin
HEK293T
C
Tet
HepAD38
D
HBV
HepG2-NTCP
E
SAMHD1
PHH
F
parental
SAMHD1-KO
DMSO
TMG
rcDNA
dsDNA
ssDNA
3.2 kb
1.6 kb
HepAD38
G
shcontrol
shOGA-1
shOGA-2
OGA
H
PUGNAc
WT
S93A

Figure S6

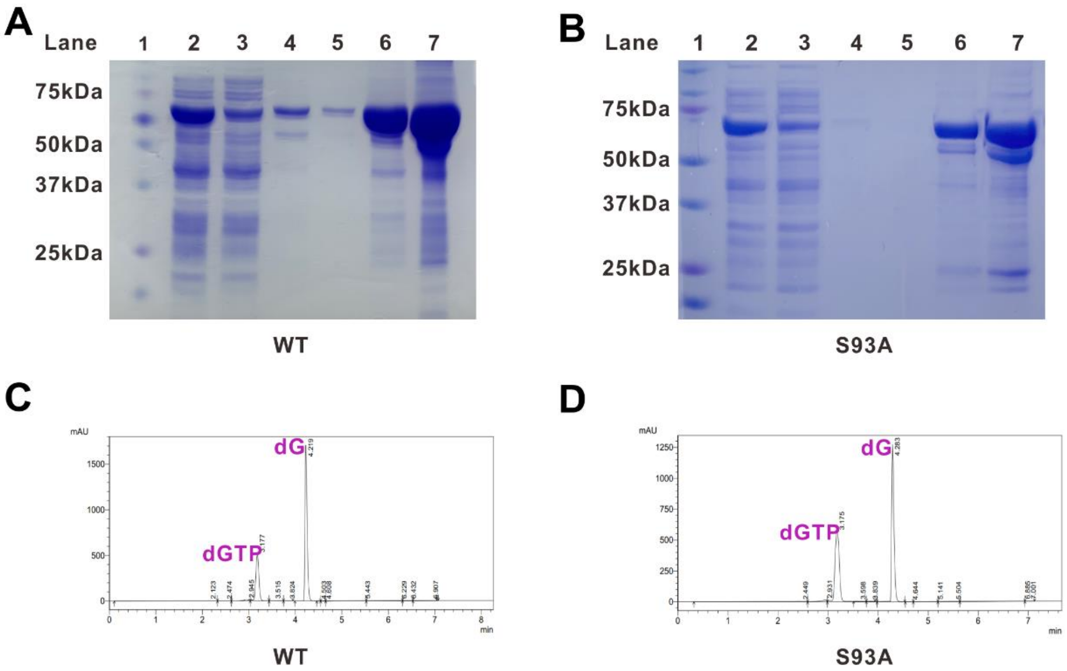
A
Lane 1 2 3 4 5 6 7
75kDa
50kDa
37kDa
25kDa
WT
B
Lane 1 2 3 4 5 6 7
75kDa
50kDa
37kDa
25kDa
S93A
C
mAU
dG
dGTP
min
WT
D
mAU
dG
dGTP
min
S93A

Table S1. Sequence of Oligonucleotides.

Name	Sequence (5’-3’)
**Primer sequences for quantitative RT-PCR.**	
HBV	Forward: TGCGGCGTTTTATCATATTCC<br>Reverse: ATACCTTGGTAGTCCAGAAGAACCA
GLUT1	Forward: ACCGCAACGAGGAGAACCG<br>Reverse: CCACAAACAGCGACACGACAG
OGA	Forward: AGCAGCCTTGAGTGGTGAGC<br>Reverse: TGGGGATTTTGATTCAGCTATG
OGT	Forward: AGAGGCAGTTCGCTTGTATCG<br>Reverse: AGTAGGCATCAGCAAAGGTAGG
GFPT1	Forward: CCAGTCCTGTCAATAGCCACC<br>Reverse: CACAAGTGCAAAAGCACCTTC
SAMHD1	Forward: TGCCCGTGTCTGTGAAGTAG<br>Reverse: ATTGCTGTAGAAATGCGATA
GAPDH	Forward: GAAATCCCATCACCATCTTCCAGG<br>Reverse: GAGCCCCAGCCTTCTCCATG
**Primer sequences for knockdown.**	
shOGA	Forward1:<br>TGGACAATTCTTTATGACATTTCAAGAGAATGTCAT<br>AAAGAATTGTCCTTTTTTC<br>Reverse1:<br>TCGAGAAAAAAGGACAATTCTTTATGACATTCTCT<br>TGAAATGTCATAAAGAATTGTCCA<br>Forward2:<br>TGCCTTTGTACACTGCGGAATTCAAGAGTTCCGC<br>AGTGTACAAAGGCTTTTTTC<br>Reverse2:<br>TCGAGAAAAAAGCCTTTGTACACTGCGGAATCTC

	TTGAATTCCGCAGTGTACAAAGGCA
shOGT	Forward1: TGCATGTTATTTGAAAGCAATTCAAGAG<br>ATTGCTTTCAAATAACATGCTTTTTTC<br>Reverse1: TCGAGAAAAAAGCATGTTATTTGAAAGC<br>AATCTCTTGAATTGCTTTCAAATAACATGCA<br>Forward2: TGCCCTAAGTTTGAGTCCAATTCAAGAG<br>ATTGGACTCAAACTTAGGGCTTTTTTC<br>Reverse2: TCGAGAAAAAAGCCCTAAGTTTGAGTC<br>CAATCTCTTGAATTGGACTCAAACTTAGGGCA
shGFTP1	Forward1: TGGAGAGAGTTATCCAACAATTCAAGAG<br>ATTGTTGGATAACTCTCTCCTTTTTTC<br>Reverse1: TCGAGAAAAAAGGAGAGAGTTATCCAA<br>CAATCTCTTGAATTGTTGGATAACTCTCTCCA<br>Forward2: TGGAAGTACTGAGCATGGATTTCAAGAG<br>AATCCATGCTCAGTACTTCCTTTTTTC<br>Reverse2: TCGAGAAAAAAGGAAGTACTGAGCATG<br>GATTCTCTTGAAATCCATGCTCAGTACTTCCA
**Primer sequences for molecular cloning.**	
pSEB-Flag-OGT	Forward: ACGCGTCGACACCATGGCGTCTTCCGTGGGC<br>Reverse: CGCGGATCCTGCTGACTCAGTGACTTCAACAGG
pBud-HA-OGT	Forward: ATAGCGGCCGCACCATGGCGTCTTCCGTGGGC<br>Reverse: CGCGGATCCTGCTGACTCAGTGACTTCAACAGG
pET28a- SAMHD1	Forward: TGCGGATCCCAGCGAGCCGATTCCGAGC<br>Reverse: CCGCTCGAGTCACATTGGGTCATCTTTAA
pSEB-FlagSAMHD1	Forward: TGAAGATCTACCATGGGCCAGCGAGCCGATTCCG<br>Reverse: CGCGTCGACCATTGGGTCATCTTTA AAAAGC
**Primer sequences for mutagenesis.**	
pSEB-Flag-<br>SAMHD1 (S93A)	Forward: ATCTTGGAGTAAGTGCCTTGGGGGAGA<br>Reverse: TCTCCCCCAAGGCACTTACTCCAAGAT

pSEB-Flag-SAMHD1 (T592E)	Forward: GGATGGCGATGTTATAGCCCCACTCATAGAACCTCAAAAA<br>Reverse: TTTTTGAGGTTCTATGAGTGGGGCTATAACATCGCCATCC
pSEB-Flag-SAMHD1 (1-150)	Forward: TGAAGATCTACCATGGGCCAGCGAGCCGATTCCG<br>Reverse: CGCGTCGACCAGCTGTTTGATGTATCGAAGAC
pSEB-Flag-SAMHD1(151-328)	Forward: TGAAGATCTACCATG GGAGGTGGTTACTATGTTTTTCC<br>Reverse: CGCGTCGACATTATTTTGGATTCCAAGATGATG
pSEB-Flag-SAMHD1(329-626)	Forward: TGAAGATCTACCATG GGCTTTGATTACAAGCGCTTTATTA<br>Reverse: CGCGTCGACCATTGGGTCATCTTTAAAAAGC
**OGT, SAMHD1-targeted single gRNAs.**	
SAMHD1- sgRNA	Forward: CACCGCTTAGTTATATCCAGCGAT<br>Reverse: AAACATCGCTGGATATAACTAAGC
OGT-sgRNA	Forward: CACCGCACATCGAGAATATCAGGC<br>Reverse: AAACGCCTGATATTCTCGATGTGC
lentiCRISPR-v2	Forward: GGTTTATTACAGGGACAGCAG<br>Reverse: ACACGACATCACTTTCCCAG

Table S2. Reagent or resource.

REAGENT or RESOURCE	SOURCE	IDENTIFIER
**Antibodies**		
Mouse anti-O-GlcNAc	Abcam	Cat#ab2739
Rabbit anti-OGT	Abcam	Cat#ab177941
Rabbit anti-OGA/MGEA5	Abcam	Cat#ab124807
Rabbit anti-GFPT1/GFAT1	Abcam	Cat#ab125069
Mouse monoclonal anti-SAMHD1	Abcam	Cat#ab67820
Rabbit anti-GLUT1	Abcam	Cat#ab115730
Rabbit anti-K48	Abcam	Cat#ab140601
Goat anti-Rabbit IgG	Abcam	Cat# ab6721
Goat anti-Mouse IgG	Abcam	Cat# ab6789
Mouse monoclonal anti-FLAG M2-peroxidase (HRP)	Sigma-Aldrich	Cat#A8592
ANTI-FLAG M2 Affinity Gel	Sigma-Aldrich	Cat#A2220
Mouse anti-GAPDH	Beyotime	Cat#AF0006
Mouse monoclonal anti-HA	Thermo	Cat#26183-HRP
Mouse anti-β-actin	ZSGB-BIO	Cat#TA-09
Rabbit polyclonal anti-HBcAg	Dako	Cat#B0586
Rabbit anti-p-GFPT1/GFAT1(Ser243)	Immuno-Biological Laboratories	Cat# JP28123
Goat anti-rabbit IgG/TRITC, secondary	ZSGB-BIO	Cat#ZF-0316
Goat anti-mouse IgG/TRITC, secondary	ZSGB-BIO	Cat#ZF-0313
Goat anti-rabbit IgG/FITC, secondary	ZSGB-BIO	Cat#ZF-0311
Goat anti-mouse IgG/ FITC, secondary	ZSGB-BIO	Cat#ZF-0312
**Bacterial and Virus Strains**		
E.coli BL21	Lab stock	
DH10B Chemically Competent E. coli	Lab stock	

pseudotyped HIV-1 single-round luciferase virus (HIV-LUC-G)	He et al., 1995	
Lenticrispr-SAMHD1	This study	
Lenticrispr-OGT	This study	
DH10B Chemically Competent E. coli	Lab stock	
AdHBV1.3	Lab stock	
**Biological Samples**		
Human liver tissue with chronic hepatitis B	The Second Affiliated Hospital, Chongqing Medical University.China	
Human serum with chronic hepatitis B	The Second Affiliated Hospital, Chongqing Medical University.China	
**Chemicals, Peptides, and Recombinant Proteins**		
Polybrene	Solarbio	Cat#H8761
PNGase F	NEB	Cat#P0704S
Fetal Bovine Serum	Gibco	Cat#A3160801
Trypsin	Gibco	Cat#12605010
RPMI1640	Gibco	Cat#11875500BT
Opti-MEM™ I Reduced Serum Medium	Gibco	Cat#31985070
DMEM/F-12	Gibco	Cat#11330500BT
DMEM	Gibco	Cat#11995500BT
MEM	Hyclone	Cat#SH30024.01
Penicillin-Streptomycin Solution	HyClone	Cat#SV30010
Lipofectamine 2000	Invitrogen	Cat#11668019
DMSO	Sigma	Cat#D2660
Phorbol 12-myristate 13-acetate (PMA)	Sigma-Aldrich	Cat#P8139
PUGNAc	Sigma-Aldrich	Cat#A7229
Cycloheximide	MCE	Cat#HY-12320

ST045849	TimTec	Cat#MFCD03308174
Thiamet G	Selleck	Cat#S7213
WZB117	Selleck	Cat#S7927
UDP-GlcNAc	BBL Life Sciences	Cat#7512-17-6
6-diazo-5-oxo-L-norleucine(Don)	Sigma-Aldrich	Cat#D2141
SuperSignal West Pico PLUS chemiluminescent Substrates	Thermo Fisher Scientific	Cat#34577
DAPI	Roche	Cat# 10236276001
Trizol	Invitrogen	Cat# 15596026
SYBR Green PCR Master Mix	BioRad	Cat#172-5121
**Critical Commercial Assays**		
PrimeScript RT reagent Kit	Takara	Cat# RR047A
TIANampVirus DNA/RNA Kit	TianGen	Cat#DP315
HBsAg ELISA KIT	Kehua	Cat#S10910113
HBeAg ELISA KIT	Kehua	Cat#20163400144
DIG high prime DNA labeling and detection starter kit II	Roche	Cat#11585614910
Dual-Glo® Luciferase Assay System	Promega	Cat#E2920
ALT ELISA kit	Mengbio	Cat#MBE1741
Elivision plus Polyer HRP (Mouse/Rabbit) IHC Kit	Maixin	Cat# KIT-9901
DAB kit	ZhongshanGolden Bridge	Cat# ZLI-9019
BCA protein assayKit	BEIJING DINGGUO	Cat# BCA02
Cell lysis buffer for Western and IP	Beyotime	Cat# P0013
**Deposited Data**		
**Experimental Models: Cell Lines**		
HEK293T	ATCC	
HepG2	ATCC	

HepAD38	Lab stock	
HepG2-NTCP	Lab stock	
THP-1	ATCC	
**Experimental Models: Organisms/Strains**		
Mouse: C57BL/6	Experimental Animal Center of Chongqing Medical University	
HBV-transgenic (HBV-Tg) mice	Xiamen University	
**Recombinant DNA**		
LentiCrisprv2	Lab stock	
pAdTrack-TO4	Dr. T-C He, University of Chicago, USA	
pBUD-HA-OGT	This paper	
pSEB-Flag-OGT	This paper	
pcDNA3-HA-SAMHD1	This paper	
pcDNA3-HA-ubiquitin	Kamitani et al., 1997	HA-Ubiquitin, Addgene plasmid, # 18712
pSEB-Flag-SAMHD1(WT)	This paper	N/A
pSEB-Flag-SAMHD1(S93A)	This paper	N/A
pSEB-Flag-SAMHD1(T592E)	This paper	N/A
pSEB-Flag-SAMHD1(1-150)	This paper	N/A
pSEB-Flag-SAMHD1(151-328)	This paper	N/A
pSEB-Flag-SAMHD1(329-626)	This paper	N/A
**Software and Algorithms**		
GraphPad Prism	GraphPad Software	https://www.graphpad.com.
CorelDRAW X7	Corel Corporation	https://www.coreldraw.com/

ImageJ	Schneider et al., 2012	https://imagej.nih.gov/ij/
Image pro plus	http://www.mediacy.com/imageproplus	N/A
